# Pleiotropic hubs drive bacterial surface competition through parallel changes in colony composition and expansion

**DOI:** 10.1371/journal.pbio.3002338

**Published:** 2023-10-16

**Authors:** Jordi van Gestel, Andreas Wagner, Martin Ackermann

**Affiliations:** 1 Department of Evolutionary Biology and Environmental Studies, University of Zürich, Zürich, Switzerland; 2 Swiss Institute of Bioinformatics, Lausanne, Switzerland; 3 Department of Environmental Systems Science, ETH Zürich, Zürich, Switzerland; 4 Department of Environmental Microbiology, Swiss Federal Institute of Aquatic Science and Technology (Eawag), Dübendorf, Switzerland; 5 Department of Microbiology and Immunology, University of California, San Francisco, San Francisco, California, United States of America; 6 Developmental Biology Unit, European Molecular Biology Laboratory, Heidelberg, Germany; 7 The Santa Fe Institute, Santa Fe, New Mexico, United States of America; 8 Stellenbosch Institute for Advanced Study (STIAS), Wallenberg Research Centre at Stellenbosch University, Stellenbosch, South Africa; Wageningen University, NETHERLANDS

## Abstract

Bacteria commonly adhere to surfaces where they compete for both space and resources. Despite the importance of surface growth, it remains largely elusive how bacteria evolve on surfaces. We previously performed an evolution experiment where we evolved distinct *Bacilli* populations under a selective regime that favored colony spreading. In just a few weeks, colonies of *Bacillus subtilis* showed strongly advanced expansion rates, increasing their radius 2.5-fold relative to that of the ancestor. Here, we investigate what drives their rapid evolution by performing a uniquely detailed analysis of the evolutionary changes in colony development. We find mutations in diverse global regulators, RicT, RNAse Y, and LexA, with strikingly similar pleiotropic effects: They lower the rate of sporulation and simultaneously facilitate colony expansion by either reducing extracellular polysaccharide production or by promoting filamentous growth. Combining both high-throughput flow cytometry and gene expression profiling, we show that regulatory mutations lead to highly reproducible and parallel changes in global gene expression, affecting approximately 45% of all genes. This parallelism results from the coordinated manner by which regulators change activity both during colony development—in the transition from vegetative growth to dormancy—and over evolutionary time. This coordinated activity can however also break down, leading to evolutionary divergence. Altogether, we show how global regulators function as major pleiotropic hubs that drive rapid surface adaptation by mediating parallel changes in both colony composition and expansion, thereby massively reshaping gene expression.

## Abstract 2

Bacteria are extraordinary surface colonizers and found on most natural surfaces; but despite this it remains unclear how they evolve on such surfaces. This study uses experimentally evolved strains of Bacillus subtilis to reveal that surface evolution is driven by pleiotropic hubs that adaptively modulate the expression of hundreds of genes.

## Introduction

Bacteria are remarkably effective surface colonizers, and they are widely found across natural surfaces. In competition for both space and resources, surface-bound bacteria can express numerous adaptations. For example, they can prevent surface detachment by secreting adhesive molecules [1], overcome surface tension by producing biosurfactants [2,3], and mitigate competition by releasing antimicrobial compounds [4,5]. These and other surface-associated adaptations are not only critical for interactions between bacteria inside microbial communities, but also for interactions between bacteria and their host [6]. For example, during chronic lung infection in cystic fibrosis patients, *Pseudomonas aeruginosa* populations evolve mucoid colonies that adhere well to the lung epithelium and that display increased antibiotic tolerance [7–9]. Even though the molecular pathways underlying bacterial surface colonization have been intensely studied in many species [10–14], it often remains elusive how bacterial populations evolve on surfaces.

**Fig 1:**
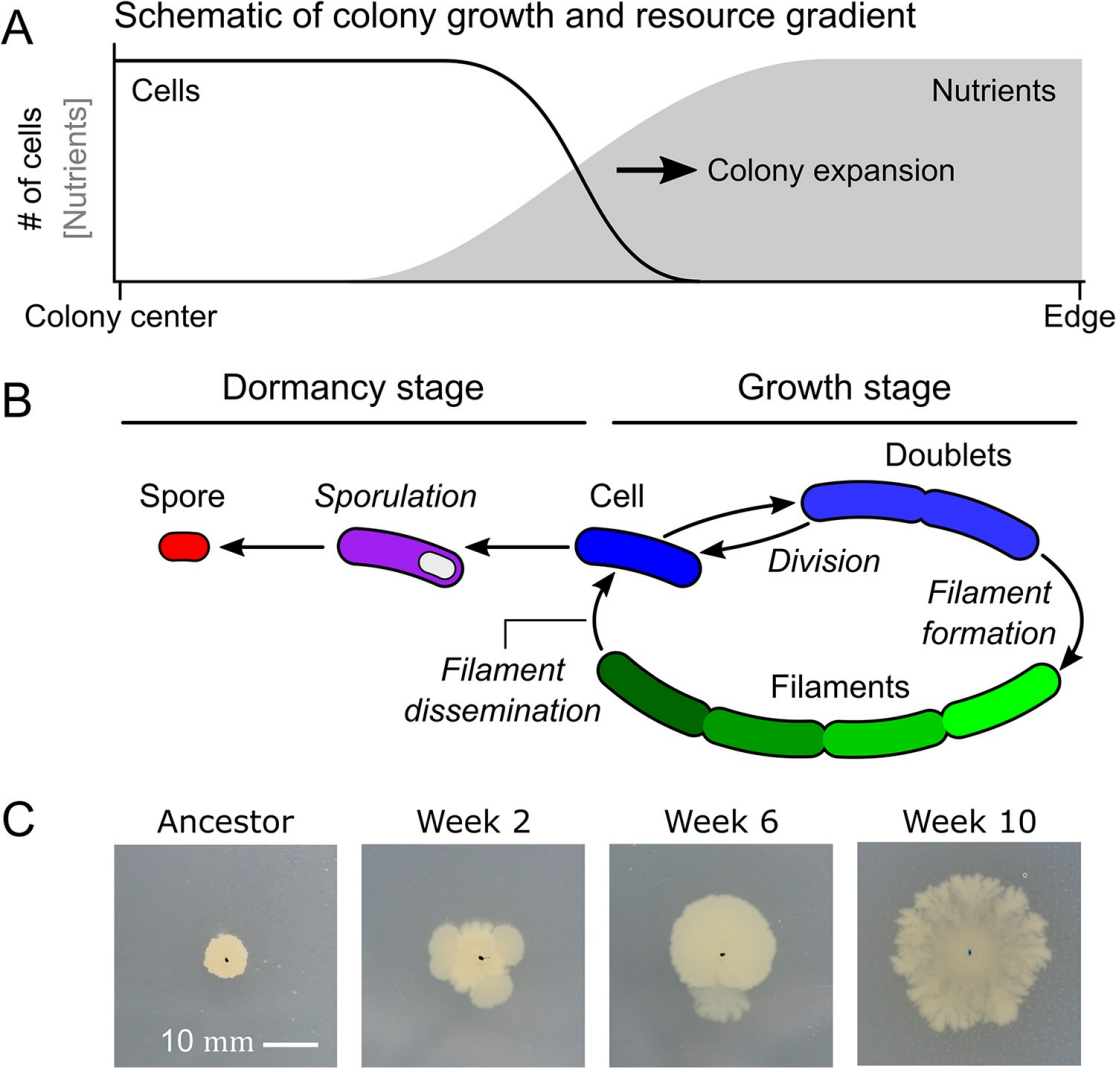
Diverse routes to evolution of advanced surface expansion. (**A**) Schematic depiction of nutrient gradients during colony expansion. Black line indicates local biomass (i.e., number of cells), and grey surface indicates nutrient concentration along a colony radius. (**B**) Schematic depiction of *Bacillus subtilis* life cycle, with a vegetative growth stage (i.e., single cells or filaments) and a dormant stage (i.e., spores). (**C**) Evolution of surface expansion observed in one lineage of the evolution experiment (*B*. *subtilis* subsp. *spizizenii* ATCC 6633).

Surface competition differs from competition during planktonic growth in that acquisition of resources is linked to surface colonization: By forming surface-bound colonies, cells can monopolize surface area and consume its associated resources [15–18]. On a surface, selection is expected to favor rapid colony expansion, leading to advanced forms of surface spreading, like bacterial gliding and sliding motility [19–21]. In contrast to planktonic growth, surface growth also results in strong spatial gradients [22], such as in resource availability, which can result in phenotypic heterogeneity [5,23–32]. Examples include colonies of the soil-dwelling bacterium *Bacillus subtilis*, where cells can be motile, form filaments, or develop spores [33,34] (Fig 1A and 1B). This phenotypic heterogeneity can directly impact surface competition [35]. For example, during sliding motility, filamentous cells are found at the colony edge where nutrients are abundant and filamentation supports expansion [21,36], while spores are mostly found in the center where nutrients are depleted and sporulation supports survival. To understand how colonies evolve, we therefore have to account for how mutations affect colony composition—e.g., the spatiotemporal expression of filamentous and sporulating cells in *B*. *subtilis*—which directly or indirectly could alter surface competition [35].

We previously studied how *Bacilli* populations evolve on surfaces using experimental evolution [37]. We exposed 8 *Bacilli* strains and species (and 4 replicates of each) to selection favoring colony spreading [38,39]: In brief, we grew colonies on a semi-solid substrate and transferred cells from the outermost colony edge to a fresh substrate every week, thereby selecting for increased rates of surface spreading. After 10 consecutive transfers, and thus 11 weeks of colony growth, we analyzed the colony size and composition for each of the 32 evolved populations [37]. Many populations evolved larger colonies and/or reduced sporulation rates. Five populations even lost the capacity to sporulate. By sequencing genomic DNA from these 5 populations, we showed that the loss of sporulation resulted from mutations that directly or indirectly inactivated Spo0A—a global regulator underlying sporulation. Yet, the loss of sporulation was not sufficient to explain the evolution of larger colonies (see also Model in S1 Text): Not all colonies that lost sporulation also increased in size, and some colonies that increased in size did not change sporulation rates.

In this study, we analyzed the populations that evolved the largest increase in colony size (approximately 2.5-fold increase in colony radius; e.g., Fig 1C) to understand what drives their rapid evolution. By focusing on a few populations only, we could comprehensively analyze which mutants arose in time and perform a uniquely detailed analysis of how these mutants affect colony development. Specifically, we quantified changes in colony expansion using automatic image analysis, changes in colony composition using high-throughput flow cytometry, and changes in gene expression using large-scale transcriptomics. By mapping quantitative changes in colony development, we uncovered how mutations in pleiotropic hubs drive adaptation by causing parallel changes in both colony composition and expansion, thereby affecting the expression of hundreds of genes.

## Results

### Mutations with pleiotropic effects on both colony expansion and composition drive adaptation

To determine what drives the evolution of rapid colony expansion, we focused our analysis on the *Bacilli* species that showed the largest increase in colony size in our evolution experiment (Fig 1C). This is *B*. *subtilis* subsp. *spizizenii* ATCC 6633 (from here onward referred to as *B*. *subtilis*) [37]. From the 4 replicate *B*. *subtilis* populations in our evolution experiment, 2 replicates increased in size the most, producing colonies with a 2.5-fold larger radius than the ancestor [37]. We concentrated most of our analysis on these 2 replicates and will refer to them as lineage 1 and 2. For comparison, we also examined the other replicate populations (lineage 3 and 4) as well as populations from *Bacillus cereus* ATCC 10987 (from here onward referred to as *B*. *cereus*), which showed the second to largest increase in colony size in our evolution experiment [37].

**Fig 2:**
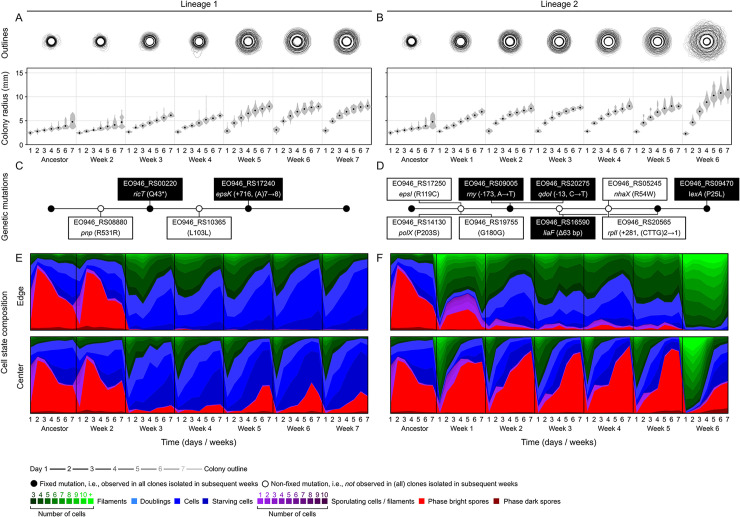
Evolution of surface expansion. Changes in colony expansion (**A**, **B**), genetic makeup (**C**, **D**), and colony composition (**E**, **F**) for clones isolated from evolved populations in the first weeks of the evolution experiment in lineage 1 (left) and 2 (right). (**A** and **B**) Colony expansion. Grey lines show colony outlines from day 1 (black outline) to day 7 (light grey outline), superimposing replicate colonies. Graph shows changes in colony radius in time (grey polygon, distribution in colony radius across replicates; black dot, mean; *n* = 3–16 colonies). Source data can be found in S1 Data. (**C** and **D**) Genetic mutations detected in clones isolated from evolved populations in lineage 1 (**C**) and 2 (**D**). Fixed mutations were observed in all clones isolated in subsequent weeks and are shown in black and nonfixed mutations were not observed in (all) clones isolated from subsequent weeks and are shown in white. For detailed overview of mutations in all isolated clones, see S3 Table. Source data can be found in S3 Data. (**E** and **F**) Colony composition at both colony edge (upper) and center (lower): filamentous cells (green), vegetative cells (blue), sporulating cells (purple), spores (red) (see figure legend). Source data can be found in S2 Data.

We systematically genotyped and phenotyped populations in lineage 1 and 2. As a starting point, we first randomly isolated 2 or 3 clones from each of our weekly archived populations from the evolution experiment. We sequenced each clone and examined colony growth dynamics (S2 Fig) by cultivating individual clones for a week and imaging their colonies daily (Methods in S5 Text). Clones showed distinct mutations (S3 Table). Some mutations were present in all clones isolated after their initial appearance. These mutations fixed in the evolved populations and are most relevant for our analysis. Other mutations did not fix and were only observed transiently. Since genotypes and growth dynamics were identical for most clones isolated from the same week (R^2^ = 0.99, *P* < 10^−16^; S2 Fig), we decided to examine the colony composition for 1 clone per week only. We did so for the first 6 or 7 weeks of our evolution experiment, where the increase in colony size was most pronounced. For each clone, we collected cells from the colony edge and center and counted the number of filamentous cells, single cells, sporulating cells, and spores, using flow cytometry (see Fig 1B and Methods in S5 Text for details). For weeks in which large changes in colony composition occurred (clones isolated from week 3 and 5 for lineage 1, and clones isolated from week 1, 2, and 6 for lineage 2; Fig 2), we also acquired transcriptomic data by collecting cells from the colony edge and/or center after 1, 2, 4, or 7 days of colony growth and performing RNA-seq (see S4 Data). Fig 2 gives an overview of the mutations that arose in lineage 1 and 2 and their effect on colony growth and composition.

In both ancestral and evolved colonies, we observed strong changes in colony composition during colony growth (Fig 2), consistent with previous studies on *B*. *subtilis* colony development [33,34]. We expect that these temporal changes in colony composition result from changes in resource gradients to which cells are exposed inside the colony (see Fig 1A and Model in S1 Text). As cells consume resources during colony growth, resource gradients emerge from the colony edge to the center [22,34], where there are more resources at the edge than in the center. This results in a relatively high fraction of spores in the center (Fig 2). Compared to previous studies [33,34], we grew colonies on a relatively poor growth medium (S1 Table) and for a long period of time (1 week), which increases the impact of resource depletion on colony development. At the colony edge, cells can however escape resource depletion by expanding outwards (S1 Text). Changes in colony composition are therefore the product of both resource consumption and colony expansion (S1 Text).

Also, over evolutionary time, we observed strong changes in colony composition, where sporulation is almost completely lost at the colony edge in both lineage 1 and 2 (Fig 2). Correspondingly, we observed a strong reduction in the expression of *spp* genes, encoding for small spore proteins [40,41], between the ancestor and evolved colonies for both lineage 1 (*log*_2_*FC* = −4.63, *P*<10^−10^; S3A Fig) and 2 (*log*_2_*FC* = −5.3, *P*<10^−7^; S3A Fig). The reduction in sporulation in lineage 1 and 2 was associated with mutations in 3 global regulators: RicT (lineage 1), RNAse Y (lineage 2), and LexA (lineage 2). The mutations affecting RicT and LexA were loss-of-function mutations (see Sections 1 and 3 in S2 Text and S5, S6, S8, and S9 Figs), while the mutation affecting RNase Y occurred upstream in the promoter region and lowered Rnase Y expression (see Section 2 in S2 Text and S5 and S7 Figs).

Each of the 3 affected global regulators have been linked to sporulation before [42–45]. RicT is part of the Y-complex, which—together with RicA and RicF—is thought to be important for regulating Rnase Y [46]. In doing so, RicT forms a stable association with Rnase Y [47]. The mutation affecting RicT in lineage 1 might thus impair sporulation through a similar mechanism than the mutation affecting Rnase Y in lineage 2. Rnase Y is one of the major endoribonucleases underlying both mRNA maturation and degradation in *B*. *subtilis* [48–50]. It was previously observed that knockouts of either *ricT* or *rny*, the genes encoding RicT and RNAse Y, cause severe sporulation defects and lower the spore frequency >100-fold [42–45] (see also S4 Fig)—which is consistent with the near loss of sporulation at the colony edge in both lineage 1 and 2 (Fig 2). It is unclear how RicT and RNAse Y exert their effect, but some evidence suggests that RicT might interfere with the phosphorylation cascade of Spo0A [43,51–55]. In lineage 2, we observed an additional mutation in LexA that led to the constitutive expression of the SOS response (Section 3 in S2 Text) [56,57]. The SOS response reduces sporulation (S4 Fig) through the Sda-dependent inhibition of Spo0A activity [58,59] and thus further lowers the sporulation rate (Fig 2F). Thus, in both lineage 1 and 2, we find mutations that directly or indirectly lower the sporulation rate, consistent with the findings in our previous study [37]. Mutants with lower sporulation rates outcompete the ancestor during colony growth, because of their higher effective growth rates (see Model in S1 Text).

Considering that the observed mutations targeted global regulators, they are expected to have many pleiotropic effects, some of which could be maladaptive. For example, RicT is important for mRNA maturation. Accordingly, we observed that our *ricT* mutant is defective in polycistronic mRNA cleavage, which might have potential negative side effects (Section 1 in S2 Text and S6 Fig). In the case of Rnase Y, it was previously shown that depletion of Rnase Y substantially increases the average mRNA half-life [48,60], which affects gene expression [49,61,62], impedes growth [45] (S4 Fig), and can even result in massive cell lysis [60]. In our experiments, we observed that the reduced *rny* expression resulted in a near loss of sporulation at the colony edge but simultaneously increased the fraction of spores in the colony center. Paradoxically, the increased fraction of spores in the colony center was not associated with a larger fraction of sporulating cells. In fact, after 3 days of colony growth, there were hardly any sporulating cells in the center (Fig 2F), while the fraction of spores peaked at approximately 80% to 90%. This suggests that the depletion of RNAse Y also resulted in cell lysis in our experiment, which led to the enrichment of spores in the colony center. Since we only transferred cells from the colony edge, cell lysis in the center would not affect colony propagation.

Pleiotropic effects can also be adaptive. This is perhaps most evident in lineage 2, where the *lexA* knockout mutant not only altered colony composition but also strongly affected colony expansion, causing an approximately 50% increase in colony radius (Fig 2B). Many bacterial species induce filamentation when activating the SOS response, which is broadly referred to as SOS filamentation [57,63]. In *B*. *subtilis*, SOS filamentation is mediated by YneA, which is suppressed by LexA [64]. Correspondingly, we observed that the *lexA* knockout mutation caused strong filamentation (Fig 2F), which enhanced colony expansion (see Section 3 in S2 Text and S9 Fig for more details). A single mutation can thus both reduce sporulation rates and increase colony expansion rates. Both phenotypes are adaptive in surface competition (S1 Text and S1B Fig).

Furthermore, the genetic mutations affecting RicT and RNAse Y have adaptive pleiotropic effects on surface expansion. It was previously shown that depletion of *ricT* and *rny* expression results in reduced expression of the *eps* operon [43,65], which underlies the production of extracellular polysaccharide (EPS). Consistently, in both lineage 1 and 2, we found significantly reduced *eps* expression in the evolved colonies compared to the ancestor (*lineage* 1: *log*_2_*FC* = −2.4, *P*<10^−8^; *lineage* 2: *log*_2_*FC* = −1.8, *P*<10^−4^; S3B Fig). EPS production mediates adhesion between cells and can thereby limit cells from expanding over a surface [49,66,67]. By reducing EPS expression, our *ricT* and *rny* mutants could therefore promote surface expansion. Indeed, beside the mutations in *ricT* and *rny* in lineage 1 and 2, we found several independent mutations in the *eps* operon across our replicate populations (*epsD*, *epsF*, *epsI*, *epsK* mutants) (S3 Table), each of which resulted in increased colony size (Section 4 in S2 Text, Figs 2 and S10). Since mutations in the *eps* operon enhance colony expansion, they allow cells to escape resource depletion, thereby lowering the fraction of sporulating cells at the colony edge (see S1 Text and S10 Fig). The impact of these mutations on sporulation is however small compared to that of the sporulation-inhibiting mutations in *ricT* and *rny* (Fig 2). Thus, like the *lexA* mutant, the *ricT* and *rny* mutants also have adaptive pleiotropic effects on both sporulation and colony expansion (Fig 2).

Besides the mutations affecting RicT, RNAse Y, and LexA, we also observed mutations with minimal or no effect on either colony size or composition (Fig 2), as detailed in Section 4 in S2 Text. These mutations might solely increase the cell division rate (see Section 4 in S2 Text and S11 Fig), with no observable consequence for both colony size and composition (see S1 Text), or they may have no phenotypic effect at all (e.g., synonymous amino acid substitutions; Fig 2). Previous studies have shown that bacterial surface expansion can result in low effective population sizes, which could promote the fixation of neutral or even maladaptive mutations [68].

Finally, for comparison, we also analyzed the evolved populations of *B*. *cereus* (S4 Table), which yielded highly similar results. *B*. *cereus* populations showed the second to largest increase in colony size during our evolution experiment [37]. Similar to our observations in *B*. *subtilis*, they harbored mutations that had pleiotropic effects on both sporulation and colony expansion (S4 Table). For example, as observed in our previous study [37], in one of the *B*. *cereus* replicate populations, there is a mutation in Spo0A, which reduces sporulation. Spo0A, however, also affects EPS production [69]. Thus, similar to the mutations affecting RNase Y and RicT in *B*. *subtilis*, the mutation in Spo0A is expected to have adaptive pleiotropic effects on both sporulation and EPS production [70–73]. In another replicate population of *B*. *cereus* (S4 Table and S12 Fig), we found 2 independent mutations affecting sporulation (in *spoVG*) and EPS production (in *epsF*). The sporulation mutant mainly affects the colony composition (S1 Text), while the EPS mutant strongly improves colony expansion (see both S1 Text and S12 Fig). Altogether, the results in *B*. *cereus* corroborate our findings in *B*. *subtilis*.

In summary, we find that bacterial surface competition favors mutations in global regulators, like RicT, Rnase Y, LexA, and Spo0A [37], with comparable pleiotropic effects on both colony size and composition: lowering the rate of sporulation and facilitating expansion by either reducing EPS production or causing filamentation. These global regulators function as pleiotropic hubs. They explain why even in our short evolution experiment we observed rapid adaptive changes in both colony size and composition (Fig 2).

### Global expression changes in evolution follow temporal expression changes during colony growth

**Fig 3:**
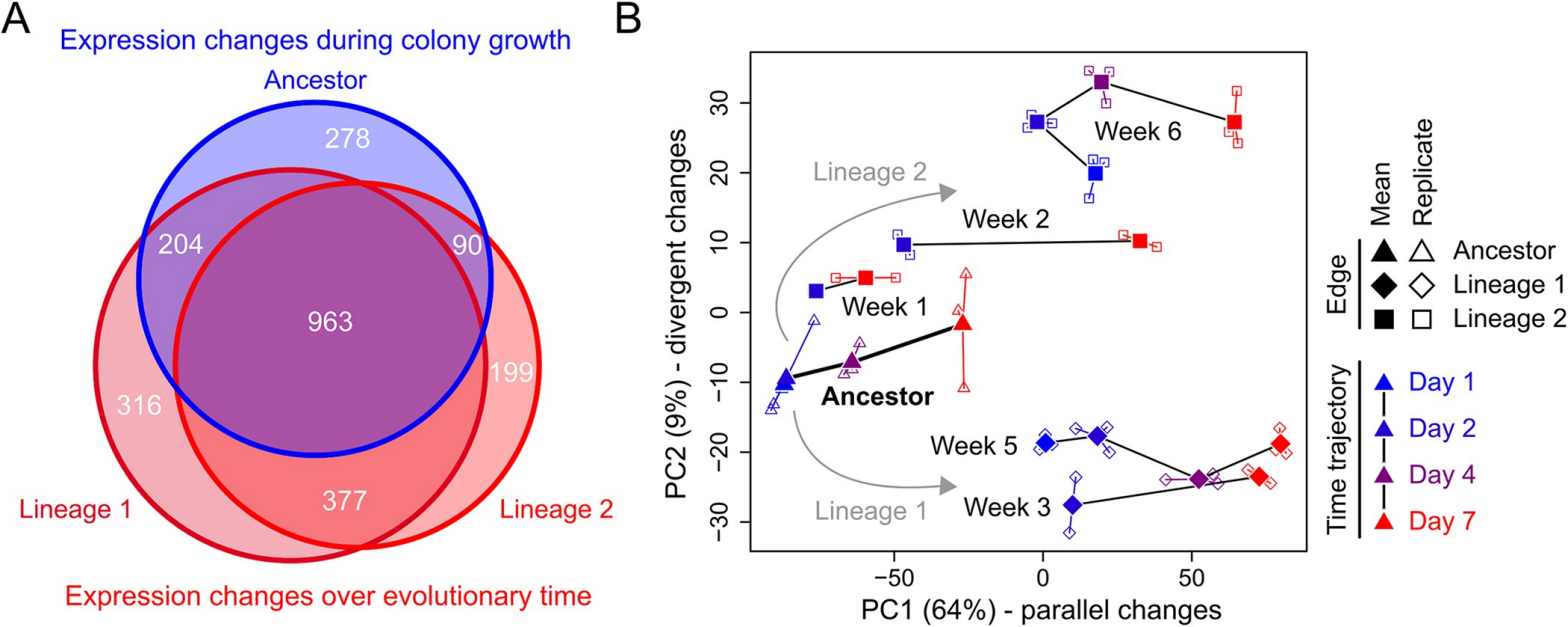
Global gene expression changes at colony edge. **(A)** Venn diagram with gene expression changes during colony growth in the ancestor and over evolutionary time: Blue circle represents all genes that significantly changed expression over a colony growth cycle in the ancestor; red circles represent all genes that significantly changed expression between the ancestral and evolved populations (see Methods in S5 Text). There is a significant overlap between the differentially expressed genes in lineage 1 (*p*<10^−16^) and lineage 2 (*p*<10^−16^). In addition, there is significant overlap between genes that evolved differential expression in lineage 1 and 2 (*p*<10^−16^) and those that changed expression over a single colony growth cycle in the ancestor. Statistics show two-sided Fisher’s exact test. (**B**) Principal component analysis of expression profiles. Time trajectories show change in expression profiles from day 1 (blue) to day 7 (red), for the ancestor (triangles) or evolved populations (lineage 1, diamonds; lineage 2, squares). Open symbols show individual replicates, and closed circles show mean expression (*n* = 3). Source data can be found in S4 and S5 Data.

Given that global regulators control the expression of many genes, we next analyzed our transcriptomic data, which revealed massive expression changes in both lineage 1 and 2. For each lineage, we compared the transcriptomes of ancestral and evolved colonies (see Methods in S5 Text) over the duration of colony growth, using either 2 or 3 replicates per time point. Approximately 45% of all genes changed expression at least 2-fold in the course of our evolution experiment (Fig 3A, S5 Data, Methods in S5 Text): 46% (1,860/4,039) in lineage 1 and 40% (1,629/4,039) in lineage 2. These massive expression changes showed strong parallelism between lineage 1 and 2, with a significant overlap (approximately 62%) of genes that change expression in both lineages (Fig 3A). Since such a large number of expression changes cannot be explained by direct regulatory targets of RicT, RNAse Y, or LexA [74–76], we hypothesized that many expression changes indirectly result from changes in colony development, which affect the conditions to which cells are exposed inside the colony and, hence, their expression. Consistent with this hypothesis, we found that most genes that change expression during colony development in the ancestor (approximately 82%), i.e., genes whose expression is sensitive to colony growth conditions, also change expression on the longer, evolutionary time scale (Fig 3A, see also S3 Text).

To further investigate the parallel expression changes, we performed a principal component analysis (PCA), which underscored the importance of temporal expression changes in explaining the parallelism between lineage 1 and 2. For this PCA, we compared all expression profiles acquired from the colony edge from both ancestral and evolved colonies, during colony growth (Fig 3B). Replicate samples showed highly reproducible expression profiles, in line with the reproducible changes in colony size and composition we described above (Figs 2, S2, and S10). The first 2 principal components (PC1 and PC2) explain approximately 73% (64% and 9%, respectively) of the observed expression variation. Despite the large number of gene expression changes, the PCA reveals a strikingly simple structure: First, expression changes that occur during colony growth are largely explained by PC1, meaning that PC1 captures temporal expression changes. Second, as colonies evolve in both lineage 1 and 2, they are projected higher on PC1 relative to the ancestor, showing that the parallel evolutionary changes in gene expression largely follow the temporal expression changes that occur during colony growth. These temporal expression changes dominate our dataset and explain most of the observed expression variation (PC1, 64%). The second principal component (PC2, 9%) captures the evolutionary divergence between lineage 1 and 2, which is minor compared to the parallel expression changes. It is in part explained by differential activity of the SOS response, which is constitutively expressed in lineage 2 only, due to the *lexA* mutation (see Section 3 in S2 Text and S3 Text).

What explains the dominant role of temporal expression changes in our dataset? We expect that cells are exposed to strong resource gradients inside the colony, which trigger the transition from vegetative growth to dormancy [33,34] (Fig 1 and S1 Text). Since some regulators are active during vegetative growth and others become activated during sporulation, changes in resource gradients will cause concordant changes in gene expression. Likewise, mutants that improve colony expansion and reduce sporulation will increase the activity of regulators underlying vegetative growth and lower those underlying dormancy. We therefore expect that the gene expression changes along PC1 are largely explained by changes in the activity of regulators underlying vegetative growth and dormancy. To test this, we first determined which of the 40 largest regulons in *B*. *subtilis* (as described by the *Subti*Wiki database [74–76]) are enriched among the set of differentially expressed genes during colony growth in the ancestor (S5 Data). We found that 18 of the 40 regulons significantly changed expression during colony growth, which included 1 phage regulon (Xpf) and 17 regular *B*. *subtilis* regulons, like those underlying sporulation [37].

To link the expression of these regulons to either vegetative growth or dormancy, we first derived the activity of their associated regulators (see Methods in S5 Text). To do so, we accounted for both the type of regulator, activator or repressor, and the expression of its target genes (S5 Text): (1) a repressor (e.g., CcpA, AbrB, SinR) is assumed to be active when its downstream genes have low expression; and (2) an activator (e.g., σ_B_, σ_D_, σ_E_, σ_F_, σ_K_, σ_G_,) is assumed to be active when its downstream genes have high expression. The activity of a regulator is not equal to its expression, because many regulators are subject to posttranscriptional regulation, such as protein sequestering [77,78] or phosphorylation [79–81]. Accordingly, we found strong correlations between the expression and activity of some regulators, like the sporulation sigma factors [82] (σ_E_, *R*^2^ = 0.94; σ_F_, *R*^2^ = 0.94; σ_K_, *R*^2^ = 0.91; σ_G_, *R*^2^ = 0.88), and much weaker correlations for others, like CodY (*R*^2^ = 0.21) and SinR (*R*^2^ = 0.09) [78,79] (S13 Fig). For most regulators, the correlation between activity and expression was in between these extremes.

**Fig 4:**
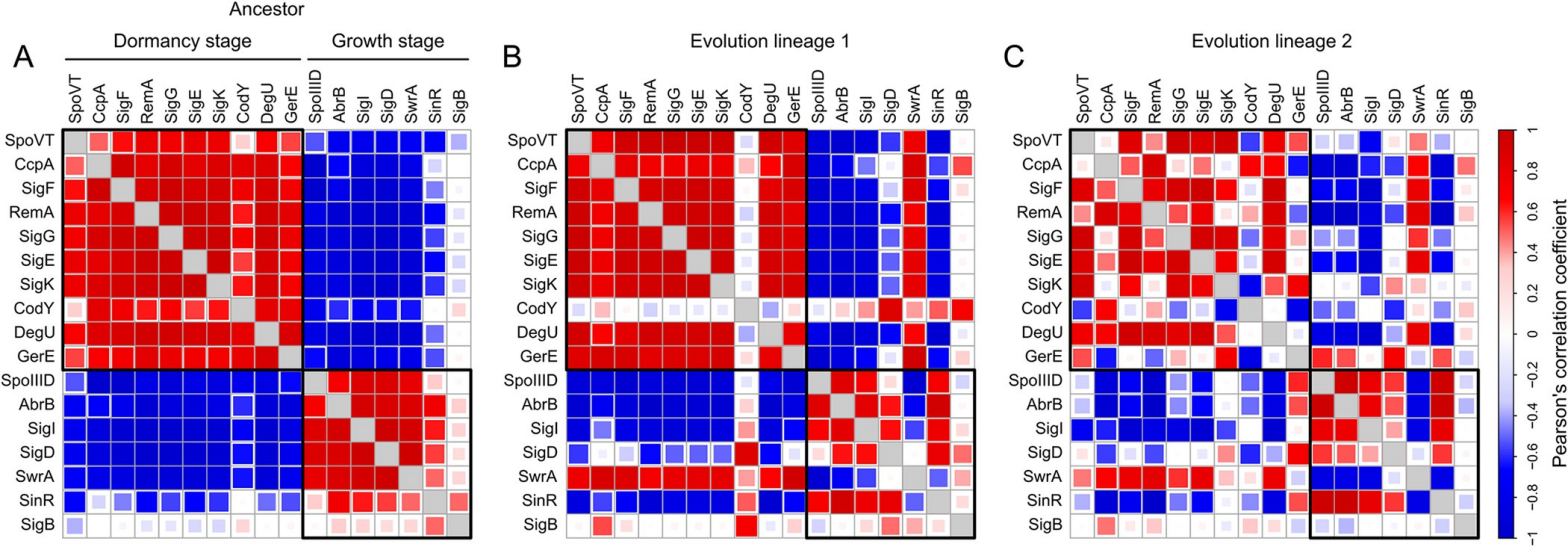
Coactivation patterns of regulators in ancestral and evolved colonies. Coactivation pattern of 17 regulators, whose regulons are enriched among genes showing significant expression changes in time. Color and size of squares show Pearson’s correlation coefficient between activities of regulators across samples of ancestral or evolved colonies (see Methods in S5 Text). Coactivation pattern is shown for the ancestor (**A**) and evolved populations of lineage 1 (**B**) and lineage 2 (**C**). Source data can be found in S4 and S5 Data.

If the transition from vegetative growth to dormancy explains the dominant role of temporal expression changes in our dataset, we expect regulatory activities to cluster in 2 modules corresponding to these life stages. Indeed, when computing pairwise correlations between the activities of all 17 regulators during colony growth in the ancestor, we found 2 clearly distinct activity modules (Fig 4A): One module of regulators is active during vegetative growth, and another module is active in the transition toward dormancy. These modules of regulators showed highly consistent changes in activity during colony growth (S14 Fig), which correlated with the fraction of sporulating cells in the colony, as determined by our flow cytometry data (*R*^2^ = 0.65±0.04, *mean*±*s*.*e*.; S15 Fig). In other words, when we observe few sporulating cells at the colony edge, based on our flow cytometry data, we also observe low activity of regulators corresponding to the dormant life stage—affecting the expression of hundreds of genes. Mutations that lower the sporulation rate likewise reduce the activity of these regulators and thus move the expression trajectories along PC1 in our PCA (Fig 3B).

The evolutionary changes in regulatory activity are also evident when studying the expression of regulons directly (S3 Text and S16–S18 Figs). For example, the regulons underlying sporulation show a coordinated decrease in expression during colony growth in both the ancestor and evolved colonies (S16 and S18 Figs). Despite the marginal fraction of sporulating cells in the evolved colonies of both lineage 1 (week 5) and 2 (week 6), we can still detect these temporal expression changes, which explains why we can also observe the parallel expression changes between the ancestral and evolve colonies in the PCA (Fig 3B). Only for lineage 2 we find a partly curved expression trajectory in Fig 3B, which is associated with temporary increase in sporulation activity during colony growth (S16 Fig).

Over evolutionary time, the modularity in regulatory activities can also break down. To compare the ancestral and evolve colonies, we examined the coactivation of regulators in the evolved colonies of lineage 1 (week 5) and 2 (week 6). Even though these colonies still showed clear modularity in regulatory activity, corresponding to vegetative growth and dormancy, they diverged from their ancestor (Fig 4B and 4C): In lineage 1, the activities of CodY, σ_D_, and σ_B_ were partly decoupled from that of the other regulators (Fig 4B), and, in lineage 2, this decoupling affected several more regulators (Fig 4C). Many of the regulators that changed activity were not directly affected by mutations. For example, the activity of σ_D_—the regulator underlying motility—became partly decoupled in both lineage 1 and 2, even though there were no direct mutations affecting motility. In the ancestor, the expression of motility genes was inversely correlated with that of genes underlying EPS production and sporulation, while in the evolved colonies, motility genes were constitutively expressed (S3 Fig).

Finally, we also examined the activities of regulators in the *B*. *cereus* lineage that only displayed targeted mutations affecting sporulation and EPS production (S12 Fig). Like for *B*. *subtilis*, also in *B*. *cereus*, we observed a strong coupling of regulatory activities in the ancestor, which became partly decoupled in the evolved colonies (S4 Text and S19 Fig). This shows that decoupling of regulatory activities does not strictly rely on the occurrence of mutations in global regulators. An exciting task for the future lies in elucidating what causes the decoupling of regulatory activities in the evolved colonies.

## Discussion

Many bacterial species form surface-bound colonies as their primary mode of living, where they evolve in competition for space and resources. In contrast, most of our experimental knowledge on bacterial evolution comes from competition experiments under planktonic growth conditions [83]. As a result, we still have a relatively poor understanding about how bacteria evolve on surfaces [16,84–88]. How do genetic mutations affect the organization of bacterial colonies and how do these organizational changes affect spatial competition? In this study, we addressed these questions by performing a uniquely detailed analysis on the evolution of surface colonization. Starting from a previous evolution experiment [37], in which we evolved *Bacilli* colonies under a selective regime that favored colony spreading, we studied the populations that evolved the largest increase in colony size. We uncovered how mutants in pleiotropic hubs drive rapid surface adaptation: lowering the rate of sporulation and promoting colony expansion. These evolutionary changes in colony development had major consequences for global gene expression, causing parallel and highly reproducible changes in the expression of nearly half of all genes.

The parallel expression changes result from the coordinated manner by which regulators change activity in time: Mutations that lower the induction of sporulation and facilitate colony expansion enhance the coordinated activity of regulators underlying vegetative growth, while suppressing those underlying dormancy. Coordinated changes in regulatory activity over both the short developmental time scale and longer evolutionary time scale also explain why just a single component in our PCA is sufficient to capture most expression changes (Fig 3B). We previously showed that transcriptomic profiles in *B*. *subtilis* obtained from vastly different environmental conditions also largely follow a single dimension, which as well corresponds to the developmental transition from vegetative growth to dormancy [37]. Our evolution experiment thus reveals that evolutionary changes in global gene expression largely follow the same dimension as well.

We expect that large-scale expression changes might commonly be observed during evolution in surface-bound bacteria: On the surface, cells are exposed to strong spatial gradients in resource availability [5,27,30]; whenever colony growth dynamics change over evolutionary time, cells will be exposed to altered resource conditions, which will affect their spatiotemporal gene expression [89]. We also observed that coactivation patterns of regulators can become decoupled over evolutionary time, leading to evolutionary divergence between populations. For example, in our study, there was a partial decoupling in regulatory activity due to consecutive activation of the SOS response in lineage 2 (Fig 4). This shows that evolution might lead to both rapid parallel changes in global gene expression as well as a slower gradual divergence. This divergence is expected to increase as more and more mutations accumulate in time.

In our study, RicT, RNAse Y, LexA, and Spo0A [37] function as pleiotropic hubs that allow for mutations that simultaneously lower the sporulation rate and improve colony expansion. These mutations thus tip the balance between growth and survival, in favor of the former. In ecology, there is a widely observed life-history tradeoff between growth and survival [90–92], and it is fascinating to see that this fundamental tradeoff also manifests itself at the level of several global regulators that mediate the expression of alternative suites of phenotypic traits. These regulators make it possible to tune the relative investment in growth and survival both over evolutionary time, in response to mutations, as well as over ecological time, in response to environmental stimuli (e.g., during colony growth).

Besides the tradeoff between growth and survival, other phenotypic tradeoffs can affect the evolution of surface colonization as well. For example, in a previous evolution experiment on swarming motility in *P*. *aeruginosa* [16], a tradeoff was observed between biofilm formation and swarming motility [16,93]. This tradeoff relates to the more widely observed tradeoff between surface colonization and dispersal [23,90,94–96]. The evolution of surface colonization might thus generally be associated with mutations in pleiotropic hubs that balance distinct phenotypic tradeoffs: In *Bacilli*, this tradeoff is evident from changes in sporulation, but tradeoffs could also be linked to cell division, stress resilience, and motility [88,97–99]. An important future challenge lies in deciphering the ecological and evolutionary implications of such phenotypic tradeoffs [99]—how does ecology shape bacterial life-history tradeoffs and how do bacteria balance these tradeoffs in response to their ecology?

Since we studied evolution under conditions that favor growth, by weekly transferring cells to a fresh growth medium, there were no adverse conditions that required sporulation. Other evolution experiments on surface colonization are performed under similar growth-promoting conditions as well [16,88]. In natural environments like the soil, periods of growth can however be interspersed by periods of adversity, which may require survival strategies like sporulation [100]. This raises the question how surface colonization evolves under more dynamical growth conditions, where prosperous and adverse conditions alternate in time. We would therefore advocate that future studies on the evolution of surface colonization should not only account for spatial complexity of a bacterium’s ecology but also its temporal complexity—in the case of *Bacilli*, this calls for more extensive efforts of bringing soil ecology into the lab [101].

## Supporting information

S1 Fig Phenomenological model on surface growth and competition. (**A**) Simulation of colony growth with changes in biomass (blue line), fraction of spores (dashed red line), and nutrient concentration along the colony radius from day 1 (bright color) to day 7 (dark color) of colony growth (S1 Text). (**B**) Model predictions on competition between wild-type and mutant genotypes that differ in (left) their biomass diffusion coefficient, (middle) their sporulation probability, and (right) their growth rate. Grey arrow indicates direction of selection. From top to bottom: percentage of mutant cells at colony edge, colony radius and percentage of spores at colony edge. See S1 Text for ordinary differential equations underlying figures.
(TIF)

S2 Fig Comparison of colony growth in replicate clones, isolated from the same week, in ancestral and evolved populations of lineage 1 and 2. In each week of the evolution experiment, for both lineage 1 and 2, we isolated 2 or 3 clones from the evolved population. Here, we compare clones with regard to colony growth. As comparison, we also compare 2 clones isolated from the ancestral population. (**A**) Comparison of colony size between clones from ancestral and evolved populations over weekly growth cycle. Solid line shows linear regression (*p*<0.05). (**B**) Change in colony size over growth cycle of ancestral clones. (**C**) Change in colony size for clones of lineage 1. Each line corresponds to 1 particular week in the evolution experiment from which the clones were extracted. (**D**) Change in colony size for clones of lineage 2. Each line corresponds to 1 particular week in the evolution experiment from which the clone was isolated. Dots show mean and error bars show standard deviation. Colors show day in the colony growth cycle (day 1 = blue; day 7 = red). Dashed line shows diagonal (i.e., colony radius the same between 2 clones isolated from the same population). Source data can be found in S1 Data.
(TIF)

S3 Fig Parallel expression changes at the colony edge in genes underlying sporulation, extracellular polysaccharide production, and motility. Relative expression of genes (normalized counts) underlying (**A**) sporulation, (**B**) extracellular polysaccharide production, and (**C**) motility in the ancestor (blue) and evolved populations of lineage 1 (upper, dark red) and lineage 2 (lower, red) at the colony edge. *n* pertains to number of genes included in the analysis. Lines show linear regressions (*p*<0.05). Note that blue data points (ancestor) are identical between upper and lower graphs and only guide as a reference to show how expression changed in the evolved populations. Source data can be found in S4 and S5 Data.
(TIF)

S4 Fig Sporulation rates and growth rates of knockout mutations in *B*. *subtilis* 168. Distribution of growth rates and sporulation rates in knockout mutations in the complete knockout library of *B*. *subtilis* 168 (data from Koo and colleagues [45]). Red, mutants observed in evolution experiment. *rny* knockout mutation does not grow in sporulation medium; therefore, growth rate is only indicated in upper histogram. Green, mutants with known negative effect on sporulation rate (*spo0A*) and positive effect on sporulation rate (*scoC*). We did observe a spo0A mutant in our evolution experiment as well. Blue, neutral markers, genes without growth or sporulation defect. Histograms show distribution of growth rates (upper) and sporulation rates (right). Source data can be found in Koo and colleagues [45].
(TIF)

S5 Fig Expression of Y-complex and RNase Y at the colony edge. Relative expression (normalized counts) of (**A**) *ricA*, (**B**), *ricF*, (**C**) *ricT*, and (**D**) *rny* in ancestor and evolved populations of lineage 1 and 2 over colony growth cycle (at the colony edge): day 1 (blue), day 7 (red). Bars, mean expression level. Statistics show two-sided Mann Whitney U test, and *p-*values are adjusted for multiple testing using Benjamini–Hochberg procedure. Source data can be found in S4 and S5 Data.
(TIF)

S6 Fig Expression of polycistronic *cggR*-*gapA* operon in ancestor and *ricT* mutant. Expression profile across *cggR-gapA* operon in (**A**) ancestor and (**B**) *ricT* mutant at day 1 of colony growth cycle. Small black dots, relative read counts per base pair; large white dot, predicted transcription starting site; blue line, estimated transcription rates; blue polygon, 95% confidence interval in estimated transcription rates, based on *Parseq* analysis [102] (see Methods in S5 Text). (**C**) Read counts of *gapA* and *cggR* in ancestor (solid circles) and *ricT* mutant (open circles) from day 1 (blue) to day 7 (red). (**D**) Expression of *cggR* relative to *gapA*. Panels show that in *ricT* mutant, *gapA-cggR* mRNA does not maturate, i.e., no mRNA cleavage, leading to near-equal expression of *cggR* and *gapA*. Source data can be found in S4 and S5 Data.
(TIF)

S7 Fig Mutations upstream of *rny* and *qdoI*. In *B*. *subtilis lineage 2*, there are spontaneous mutations upstream of both *rny* and *qdoI*. (**A**) The mutation upstream of *rny* targets the -10 element (green) just before the transcription starting site (TSS, purple), as determined by Rend-seq by DeLoughery and colleagues [46], and is expected to lower *rny* expression. Indeed, we observe a significant reduction in *rny* expression in our RNA-seq data (*log*_2_*FC* = −3.0, *P*<10^−16^, S5 Data). (**B**) The mutation upstream of *qdoI* targets the ribosomal binding site (RBS, i.e., Shine–Dalgarno sequence; blue) and is therefore expected to affect the translation rate without changing mRNA expression of *qdoI*. The “wild-type” nucleotides that are substituted in the mutants are shown in bold (asterisk). Protein coding sequences are shown in red with amino acids as red letters. The -10 and -35 promoter elements are shown in green. For the *rny* promoter, this corresponds to a conical σ_A_ promoter. The ribosomal binding sites are shown in blue. Source data can be found in S3 Data.
(TIF)

S8 Fig Mutation and expression changes in *lexA* and *yneA*. (**A**) Mutation in *lexA* (P25L) observed in lineage 2 is physically close to active binding site of LexA protein to DNA (here shown for one of the monomers). The protein structure was obtained from the Protein Data Bank RCSB PDB [103] (https://www.rcsb.org/3d-view/3k3r). Expression of (**B**) *lexA* and (**C**) *yneA* in ancestral and evolved populations over colony growth cycle: day 1 (blue) to day 7 (red). Bars show average expression (*n* = 3). Statistics show two-sided Mann Whitney U test, and *p*-values are adjusted for multiple testing using Benjamini–Hochberg procedure. Source data can be found in S4 and S5 Data.
(TIF)

S9 Fig SOS filamentation causes strongly advanced colony expansion in *lexA* knockout mutant. (**A**) Colony expansion and (**B**) colony composition in (**C**) *lexA* and *yneA* mutants in week 5 to 7 of the evolution experiment in lineage 2. The *lexA* knockout mutation in week 6 leads to SOS filamentation, mediated by the expression of *yneA* that inhibits cell division. Colony expansion is reduced in *yneA* mutant that appears in week 7, suggesting that SOS filamentation largely explains the strongly improved colony expansion rate in week 6. Since there is no selective benefit for the *yneA* mutation, as it harms colony spreading, it does not fixate in the population. For figure legend, see caption of Fig 2. Source data can be found in S1 and S2 Data.
(TIF)

S10 Fig Effect of *eps* mutations on colony expansion and composition. Colony growth in ancestor and *eps* mutants of lineage 2 and 3. Although lineage 2 and 3 acquired distinct mutations (**B**) in the *eps* operon (S3 Table), they show strongly parallel changes in colony expansion (**A**) and colony composition (**C**). For figure legend, see caption of Fig 2. Source data can be found in S1 and S2 Data.
(TIF)

S11 Fig Expression of *liaI* and *liaH* in ancestral and evolved populations. Relative expression (normalized counts) of (**A**) *liaI* and (**B**) *liaH* in ancestral and evolved population over colony growth cycle: day 1 (blue) to day 7 (red). Bars show average expression (*n* = 3). Statistics show two-sided Mann Whitney U test, and *p*-values are adjusted for multiple testing using Benjamini–Hochberg procedure. Source data can be found in S4 and S5 Data.
(TIF)

S12 Fig Evolution of surface expansion in *Bacillus cereus*. Changes in colony expansion (**A**), genetic makeup (**B**), and colony composition (**C**) during the first weeks of the evolution experiment in *B*. *cereus* (see lineage 1 in S4 Table). (**A**) Grey lines show colony outlines from day 1 (dark grey outline) to day 7 (light grey outline), superimposing different replicate colonies. Graph shows changes in colony radius in time (grey polygon, distribution in colony radius across replicates; black dot, mean; *n* = 4−14). Source data can be found in S1 Data. (**B**) Genetic mutations: with mutations that fixed in the population (black) and those that transiently appeared (white). Source data can be found in S3 Data. (**C**) Colony composition at both colony edge (upper) and center (lower): filamentous cells (green), vegetative cells (blue), sporulating cells (purple), spores (red) (for more details, see legend in Fig 2). Source data can be found in S2 Data.
(TIF)

S13 Fig Relation between expression and activity of global regulators. Relation between relative expression and activity for each of the 17 global regulators studied in Fig 4. Lines show linear regression (*p*<0.05). For some regulators (CcpA, RemA, SpoIIID, and SinR), there is a negative correlation between gene expression and regulatory activity, which could either indicate that there is posttranscriptional feedback that inhibits protein activity upon high expression or that the annotation of regulon is partially incomplete. Source data can be found in S4 and S5 Data.
(TIF)

S14 Fig Change in regulatory activity over colony growth cycle for ancestral and evolved colonies. Horizontal black line, reference that indicates no change in activity. Ancestor, blue dots and regression. Evolved population: dark red, lineage 1 and red, lineage 2. Lines show linear regression (*p*<0.05). Source data can be found in S4 and S5 Data.
(TIF)

S15 Fig Correlation between fraction of sporulating cells and regulatory activity. Relation between fraction of sporulating cells and regulatory activity for each of the 17 global regulators in Fig 4: including data from ancestral population (blue) to evolved populations (red). All regulators show strong correlation, except for CodY and SpoIIID. Lines show linear regression (*p*<0.05). Source data can be found in S4 and S5 Data.
(TIF)

S16 Fig Expression map. (**A**) Expression map of genes, based on gene expression profiles in ancestor and evolved colonies, following Kohonen’s self-organizing map. Circles show genes. Hexagons show meta-genes. Genes from the same operon frequently belong to the same meta-gene (S17 Fig). Meta-genes belonging to the same regulon cluster together, as shown for the sporulation sigma factor regulons, AbrB regulon, LexA regulon, SigB regulon, CcpA regulon, SigD regulon. (**B**) Relative expression of meta-genes at day 1, 2, 4, and 7 of colony growth in ancestral or evolved populations of lineage 1 (week 5) and 2 (week 6): blue, low expression; white, mean expression; red, high expression). Source data can be found in S4 and S5 Data.
(TIF)

S17 Fig Genes that are part of the same meta-gene in the expression map often belong to the same operon. (**A**) Expected (red) and observed (blue) distance between genes associated with the same meta-gene, calculated by the number of base pairs between their respective start codons on either the positive strand (left) or negative strand (right). (**B**) Operon diversity among genes belonging to same meta-gene. Operon diversity is calculated using a relative Shannon index: 0 indicates that all genes associated with the same meta-gene also belong to the same operon, and 1 indicates that none of the genes associated with the same meta-gene belong to the same operon. Bars show strong enrichment for low operon diversity, indicating that genes associated with the same meta-gene often belong to the same operon. This enrichment is supported by the results in (**A**), since—by necessity—genes in the same operon are in close physical proximity on the chromosome. (**C**) Converse analysis, meta-gene diversity among genes belonging to the same operon. Meta-gene diversity is calculated using a relative Shannon index: 0 indicates that all genes from the same operon are associated with the same meta-gene, and 1 indicates that none of the genes from the same operon are associated with the same meta-gene. Bars show strong enrichment for low meta-gene diversity, indicating that genes from the same operon are often associated with same meta-gene. This enrichment logically follows from the results in (**B**). Source data can be found in S4 and S5 Data.
(TIF)

S18 Fig Composition of expression map. **(A)** Overview of expression map as shown in S16A Fig. (**B**) Standard deviation in meta-gene expression across expression profiles (i.e., log-fold changes): from low (blue) to high (red) variability in expression. (**C**) Loading of meta-genes on principal component 1, 2, and 3: from low loading (blue) to high loading (red). (**D**) Fraction of genes within meta-gene belonging to functional category as defined by the *Subti*Wiki database [74–76]: motility and chemotaxis, metabolism, carbon metabolism, nucleotide metabolism, information processing, regulation of gene expression, proteins of unknown function, biofilm formation, general stress response, and sporulation. (**E**) Fraction of genes within meta-gene belonging to a particular regulon as defined by the *Subti*Wiki database [74–76]: SigD regulon, CcpA regulon, LexA regulon, SigB regulon, AbrB regulon, SigE regulon, SigK regulon, SigF regulon, SigG regulon, and GerE regulon. In (**D**) and (**E**), blue indicates that none of the genes associated with meta-gene belong to functional category/regulon; red indicates that all genes belong to functional category/regulon. White number indicates fraction of genes belonging to functional category/regulon. Source data can be found in S4 and S5 Data.
(TIF)

S19 Fig Coactivity of global regulators in ancestor and evolved population of *B*. *cereus*. Color and size of squares show Pearson’s correlation coefficient between activity of regulators in ancestral population (**A**) and evolved population (**B**). Coactivation pattern is strongly disrupted in evolved population relative to that observed in the ancestor. Source data can be found in S4 and S5 Data.
(TIF)

S20 Fig Colony image analysis. We designed a custom-made image analysis software in Matlab to determine the colony outlines in 1,388 colony images. The general user interface is shown in (**C**). First, the software automatically detects the Petri dish, using a simple segmentation procedure (**B**). Segmentation occurs through a few simple steps, which associated parameter settings can be optimized, by displaying the segmentation procedure in (**A**). After detecting the Petri dish (**B**), the colony outline is automatically detected (**C**) using another simple segmentation procedure, whose parameters could be optimized. To refine the colony boundary, small perpendicular lines are drawn along the colony outline; see yellow lines in (**C**) and (**D**), along which the pixel intensity values are measured. These values are expected to fall along a sigmoidal curve (red lines in (**E**) and (**F**)). In the refinement step, the outline (vertical blue solid line in (**E**)) is optimized such that it falls at the inflection point of the sigmodal curve (vertical blue dotted line in (**F**)). Panels (**E**) and (**F**) show how the boundary is refined at a single point along the colony outline. This procedure is done for the entire outline. The refinement step assures that the colony outline is perfectly placed, despite local differences in light intensities (i.e., due to shading or otherwise). For the rare cases where automatic refinement fails, one can also manually adjust the outline, by zooming in (as shown in (**D**)), and then removing, moving, or adding points along the outline. For a full description of the image analysis software as well as the Matlab script, see our Github repository https://github.com/jordivangestel/PLoS-Biology-2023.
(TIF)

S1 Text Model on surface growth. (PDF)

S2 Text Extended evaluation of mutations in lineage 1 and 2. (PDF)

S3 Text Expression maps. (PDF)

S4 Text Coactivation patterns in *B*. *cereus*. (PDF)

S5 Text Methods. (PDF)

S1 Table Chemically defined growth medium. (PDF)

S2 Table Strains. (PDF)

S3 Table Mutations in *B*. *subtilis* subsp. *spizizenii* ATCC 6633. (PDF)

S4 Table Mutations in *B*. *cereus* ATCC 10987. (PDF)

S1 Data Overview of image data. (CSV)

S2 Data Overview of flow cytometry data. (CSV)

S3 Data Overview of genomic sequencing data. (CSV)

S4 Data Overview of transcriptomic sequencing data. (CSV)

S5 Data Overview of transcriptomic analyses. (XLSX)
