# Supplementary figures and images for: Pleiotropic hubs drive bacterial surface competition through parallel changes in colony composition and expansion
Source: PLoS Biol. 2023 Oct 16;21(10):e3002338. doi: 10.1371/journal.pbio.3002338 (PMC10578586; doi:10.1371/journal.pbio.3002338)

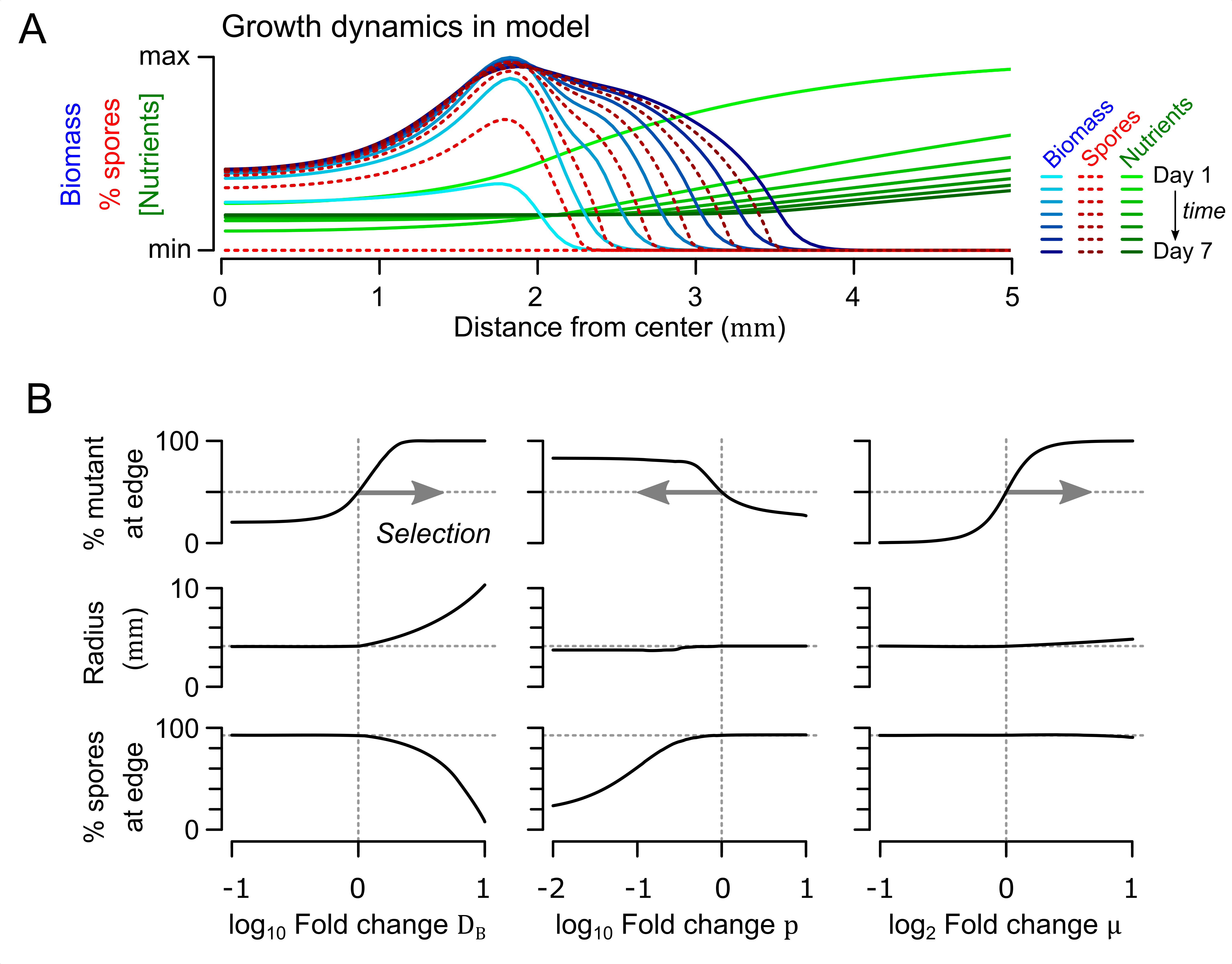

Supplement: S1 Fig — (A) Simulation of colony growth with changes in biomass (blue line), fraction of spores (dashed red line), and nutrient concentration along the colony radius from day 1 (bright color) to day 7 (dark color) of colony growth (S1 Text). (B) Model predictions on competition between wild-type and mutant genotypes that differ in (left) their biomass diffusion coefficient, (middle) their sporulation probability, and (right) their growth rate. Grey arrow indicates direction of selection. From top to bottom: percentage of mutant cells at colony edge, colony radius and percentage of spores at colony edge. See S1 Text for ordinary differential equations underlying figures. (TIF) [file pbio.3002338.s001.tif]

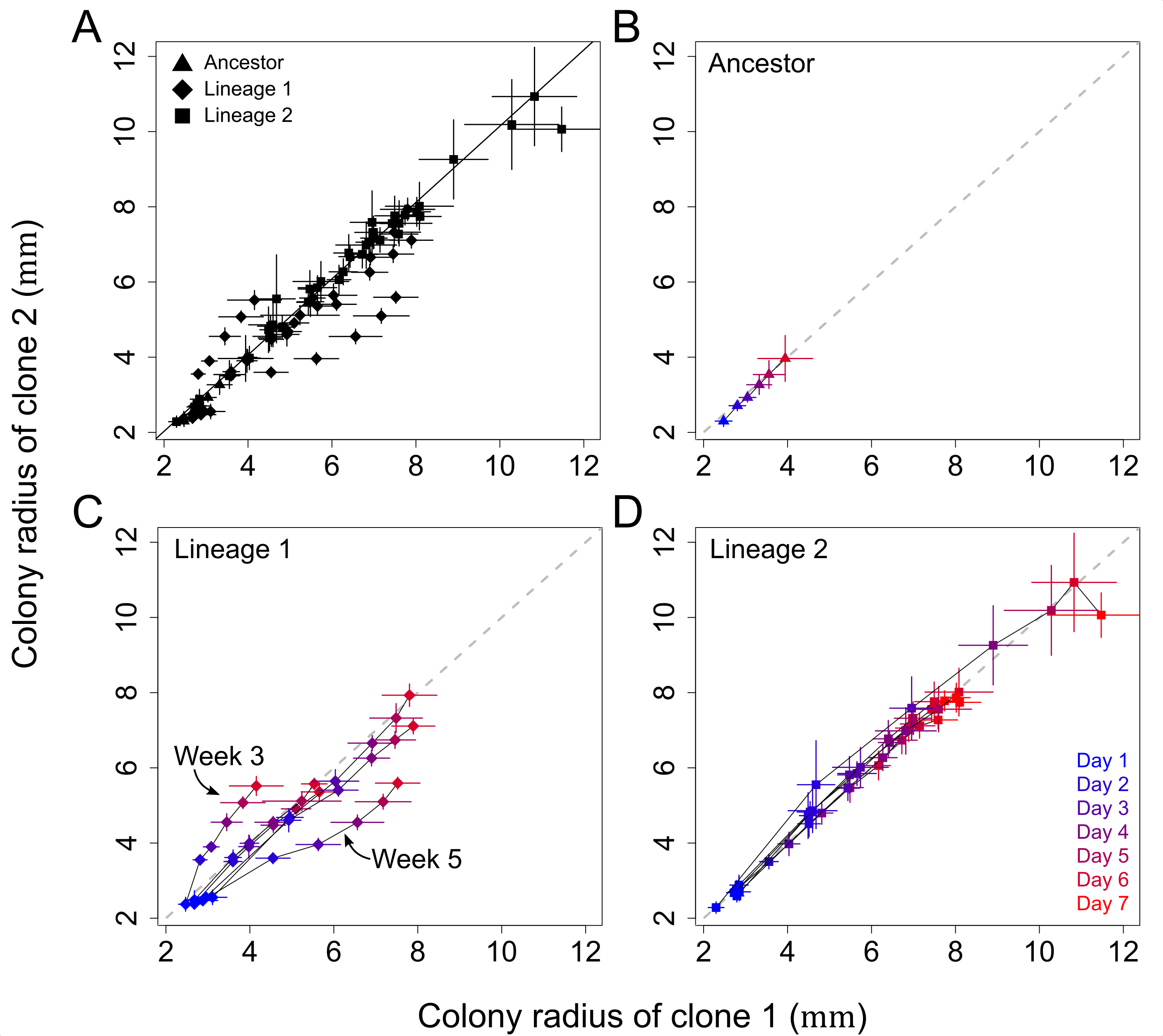

Supplement: S2 Fig — In each week of the evolution experiment, for both lineage 1 and 2, we isolated 2 or 3 clones from the evolved population. Here, we compare clones with regard to colony growth. As comparison, we also compare 2 clones isolated from the ancestral population. (A) Comparison of colony size between clones from ancestral and evolved populations over weekly growth cycle. Solid line shows linear regression (p<0.05). (B) Change in colony size over growth cycle of ancestral clones. (C) Change in colony size for clones of lineage 1. Each line corresponds to 1 particular week in the evolution experiment from which the clones were extracted. (D) Change in colony size for clones of lineage 2. Each line corresponds to 1 particular week in the evolution experiment from which the clone was isolated. Dots show mean and error bars show standard deviation. Colors show day in the colony growth cycle (day 1 = blue; day 7 = red). Dashed line shows diagonal (i.e., colony radius the same between 2 clones isolated from the same population). Source data can be found in S1 Data. (TIF) [file pbio.3002338.s002.tif]

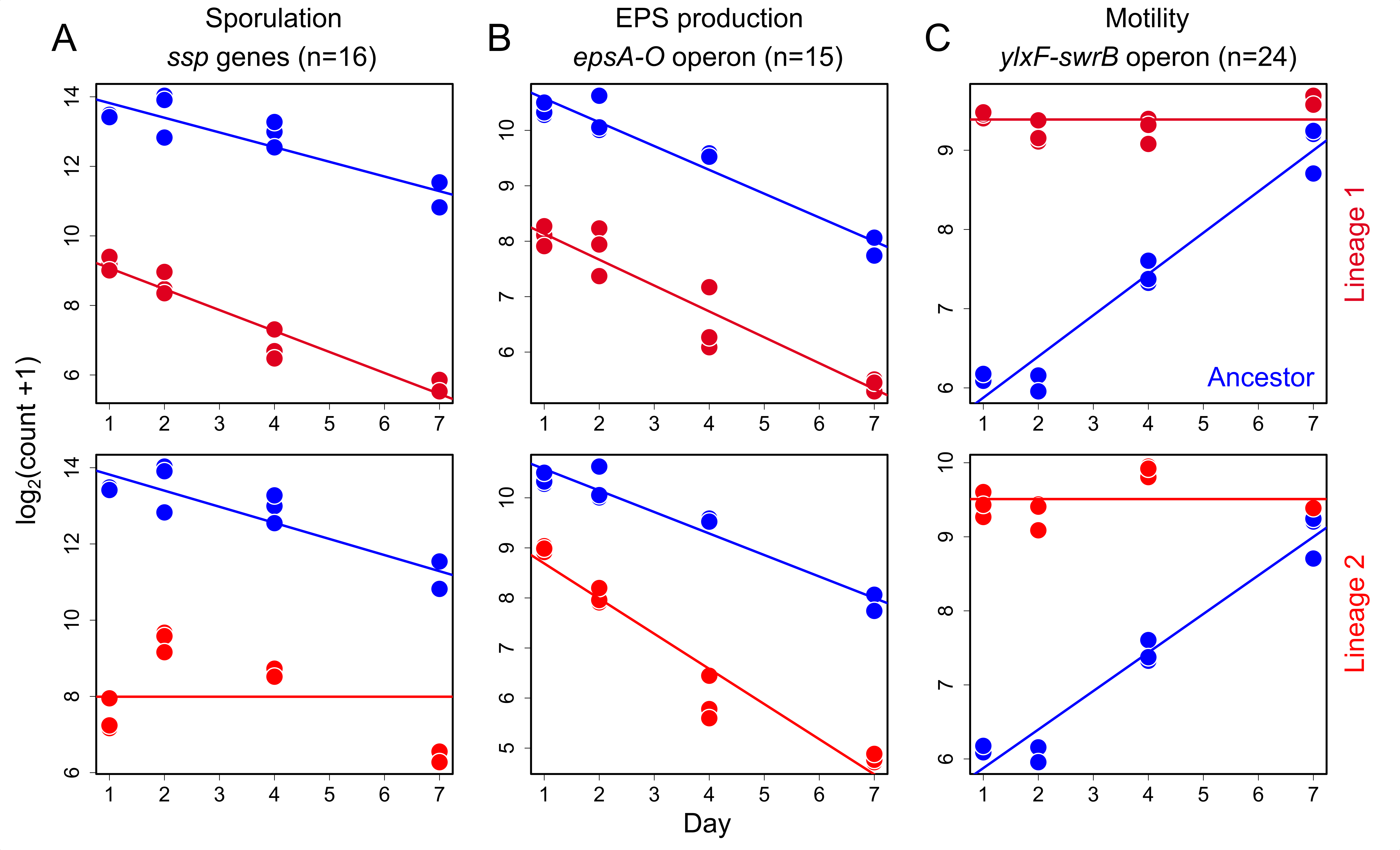

Supplement: S3 Fig — Relative expression of genes (normalized counts) underlying (A) sporulation, (B) extracellular polysaccharide production, and (C) motility in the ancestor (blue) and evolved populations of lineage 1 (upper, dark red) and lineage 2 (lower, red) at the colony edge. n pertains to number of genes included in the analysis. Lines show linear regressions (p<0.05). Note that blue data points (ancestor) are identical between upper and lower graphs and only guide as a reference to show how expression changed in the evolved populations. Source data can be found in S4 and S5 Data. (TIF) [file pbio.3002338.s003.tif]

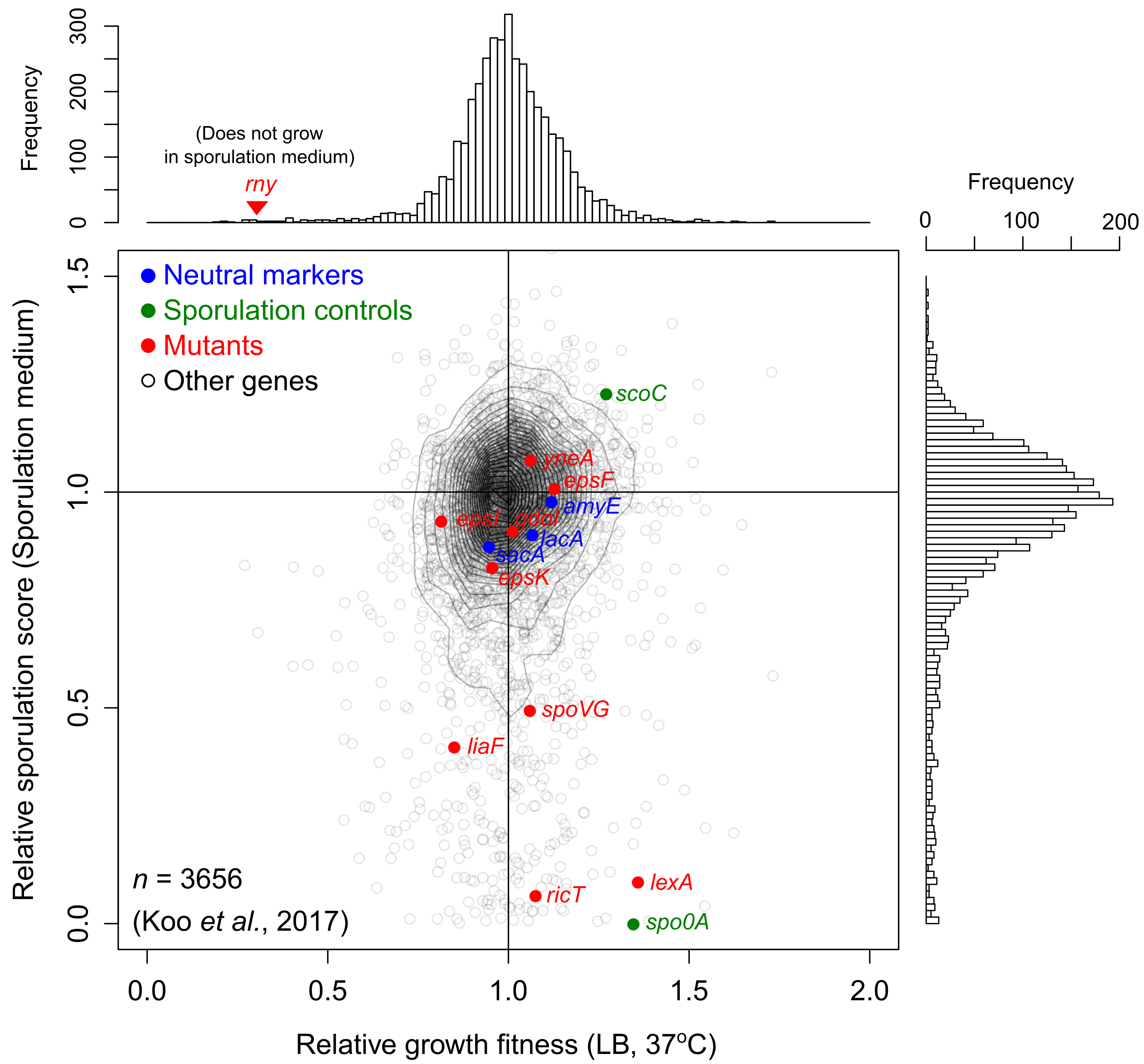

Supplement: S4 Fig — Distribution of growth rates and sporulation rates in knockout mutations in the complete knockout library of B. subtilis 168 (data from Koo and colleagues [45]). Red, mutants observed in evolution experiment. rny knockout mutation does not grow in sporulation medium; therefore, growth rate is only indicated in upper histogram. Green, mutants with known negative effect on sporulation rate (spo0A) and positive effect on sporulation rate (scoC). We did observe a spo0A mutant in our evolution experiment as well. Blue, neutral markers, genes without growth or sporulation defect. Histograms show distribution of growth rates (upper) and sporulation rates (right). Source data can be found in Koo and colleagues [45]. (TIF) [file pbio.3002338.s004.tif]

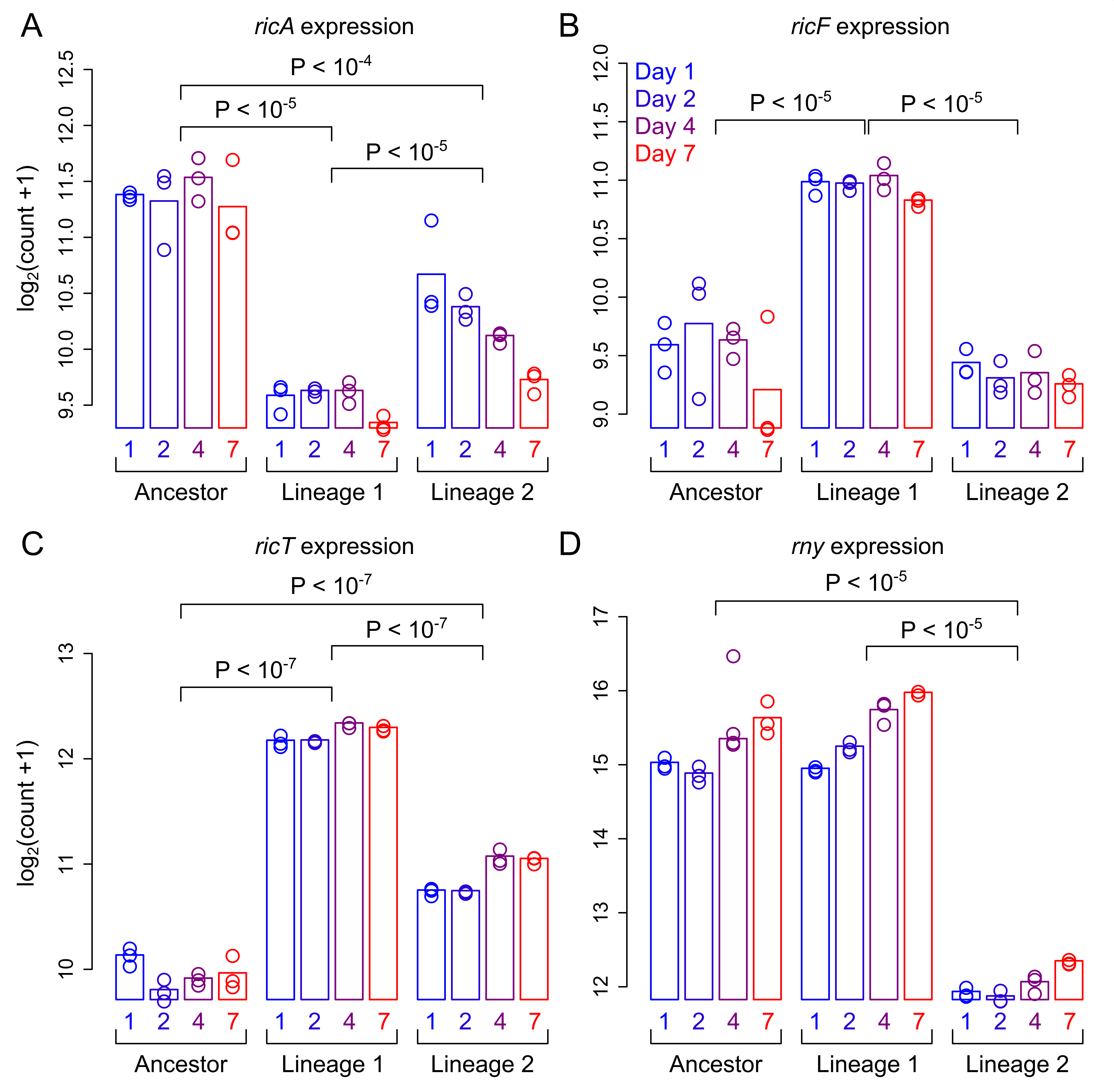

Supplement: S5 Fig — Relative expression (normalized counts) of (A) ricA, (B), ricF, (C) ricT, and (D) rny in ancestor and evolved populations of lineage 1 and 2 over colony growth cycle (at the colony edge): day 1 (blue), day 7 (red). Bars, mean expression level. Statistics show two-sided Mann Whitney U test, and p-values are adjusted for multiple testing using Benjamini–Hochberg procedure. Source data can be found in S4 and S5 Data. (TIF) [file pbio.3002338.s005.tif]

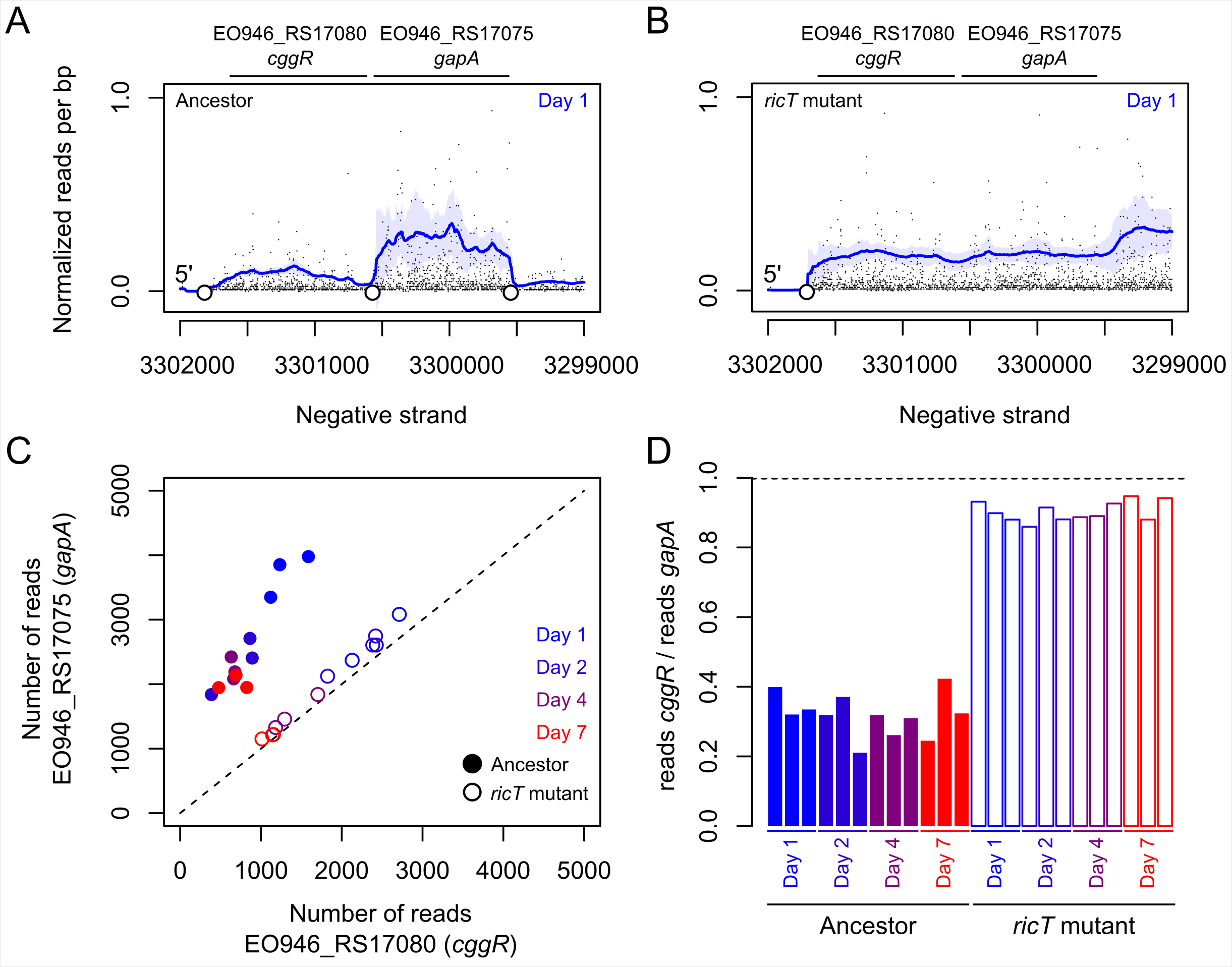

Supplement: S6 Fig — Expression profile across cggR-gapA operon in (A) ancestor and (B) ricT mutant at day 1 of colony growth cycle. Small black dots, relative read counts per base pair; large white dot, predicted transcription starting site; blue line, estimated transcription rates; blue polygon, 95% confidence interval in estimated transcription rates, based on Parseq analysis [102] (see Methods in S5 Text). (C) Read counts of gapA and cggR in ancestor (solid circles) and ricT mutant (open circles) from day 1 (blue) to day 7 (red). (D) Expression of cggR relative to gapA. Panels show that in ricT mutant, gapA-cggR mRNA does not maturate, i.e., no mRNA cleavage, leading to near-equal expression of cggR and gapA. Source data can be found in S4 and S5 Data. (TIF) [file pbio.3002338.s006.tif]

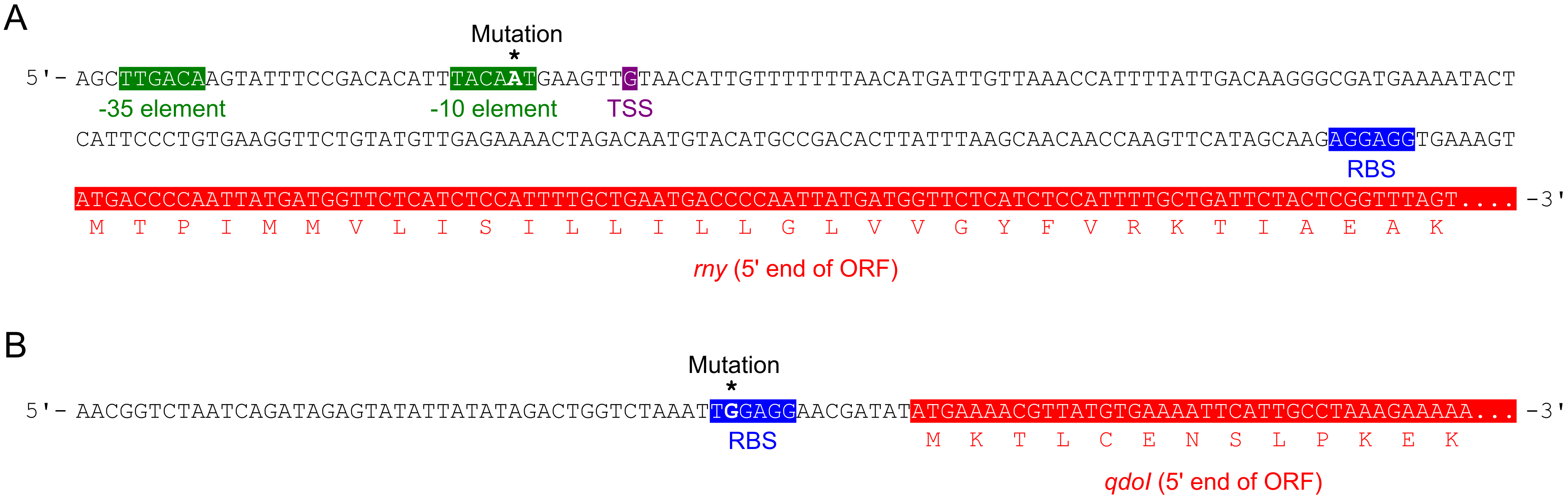

Supplement: S7 Fig — In B. subtilis lineage 2, there are spontaneous mutations upstream of both rny and qdoI. (A) The mutation upstream of rny targets the -10 element (green) just before the transcription starting site (TSS, purple), as determined by Rend-seq by DeLoughery and colleagues [46], and is expected to lower rny expression. Indeed, we observe a significant reduction in rny expression in our RNA-seq data (log2FC = −3.0, P<10−16, S5 Data). (B) The mutation upstream of qdoI targets the ribosomal binding site (RBS, i.e., Shine–Dalgarno sequence; blue) and is therefore expected to affect the translation rate without changing mRNA expression of qdoI. The “wild-type” nucleotides that are substituted in the mutants are shown in bold (asterisk). Protein coding sequences are shown in red with amino acids as red letters. The -10 and -35 promoter elements are shown in green. For the rny promoter, this corresponds to a conical σA promoter. The ribosomal binding sites are shown in blue. Source data can be found in S3 Data. (TIF) [file pbio.3002338.s007.tif]

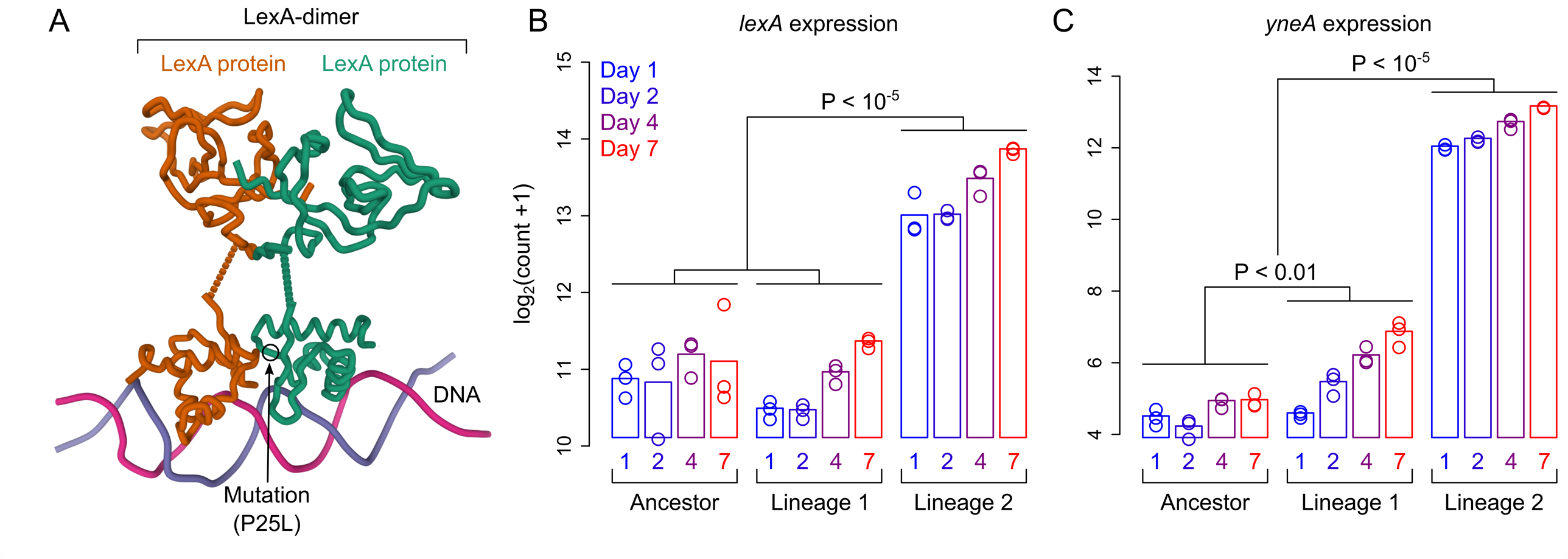

Supplement: S8 Fig — (A) Mutation in lexA (P25L) observed in lineage 2 is physically close to active binding site of LexA protein to DNA (here shown for one of the monomers). The protein structure was obtained from the Protein Data Bank RCSB PDB [103] (https://www.rcsb.org/3d-view/3k3r). Expression of (B) lexA and (C) yneA in ancestral and evolved populations over colony growth cycle: day 1 (blue) to day 7 (red). Bars show average expression (n = 3). Statistics show two-sided Mann Whitney U test, and p-values are adjusted for multiple testing using Benjamini–Hochberg procedure. Source data can be found in S4 and S5 Data. (TIF) [file pbio.3002338.s008.tif]

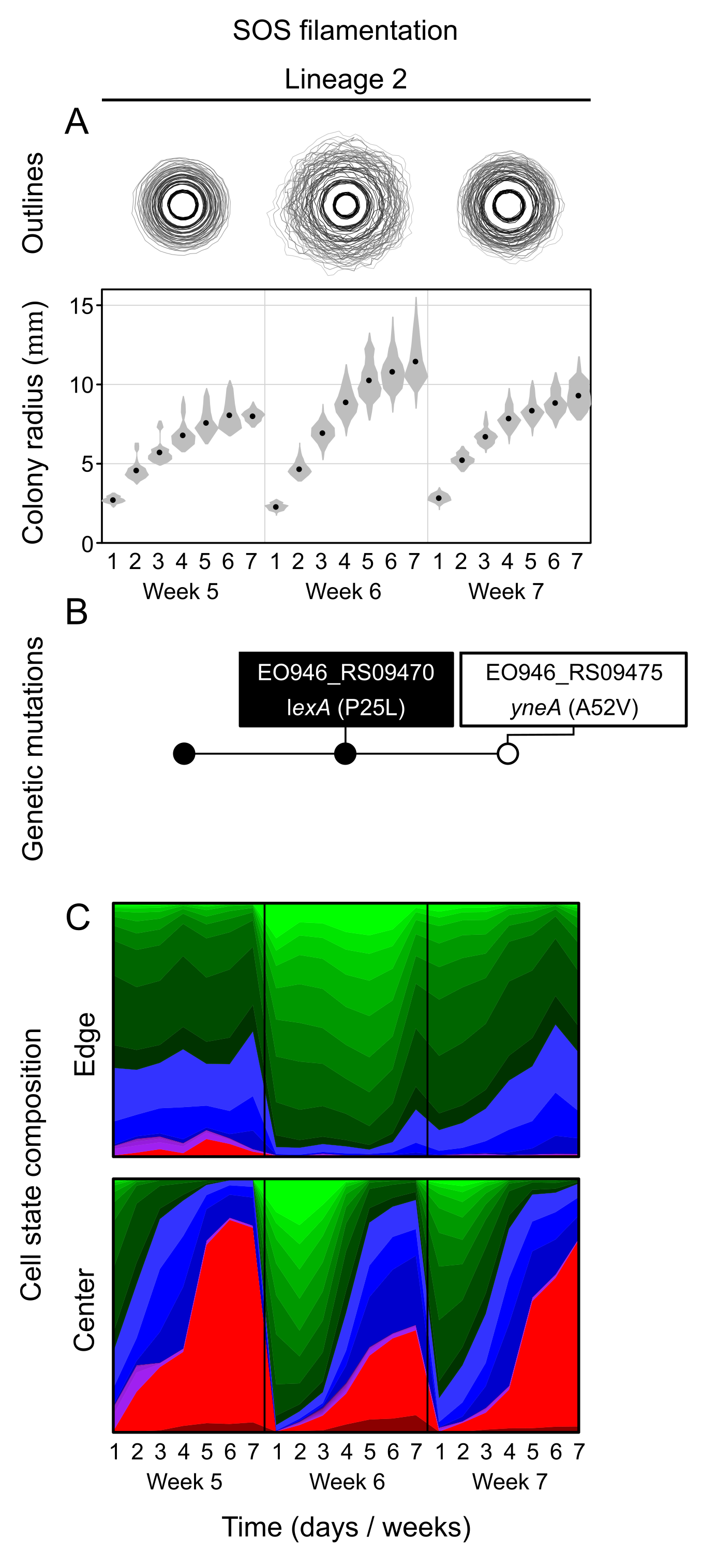

Supplement: S9 Fig — (A) Colony expansion and (B) colony composition in (C) lexA and yneA mutants in week 5 to 7 of the evolution experiment in lineage 2. The lexA knockout mutation in week 6 leads to SOS filamentation, mediated by the expression of yneA that inhibits cell division. Colony expansion is reduced in yneA mutant that appears in week 7, suggesting that SOS filamentation largely explains the strongly improved colony expansion rate in week 6. Since there is no selective benefit for the yneA mutation, as it harms colony spreading, it does not fixate in the population. For figure legend, see caption of Fig 2. Source data can be found in S1 and S2 Data. (TIF) [file pbio.3002338.s009.tif]

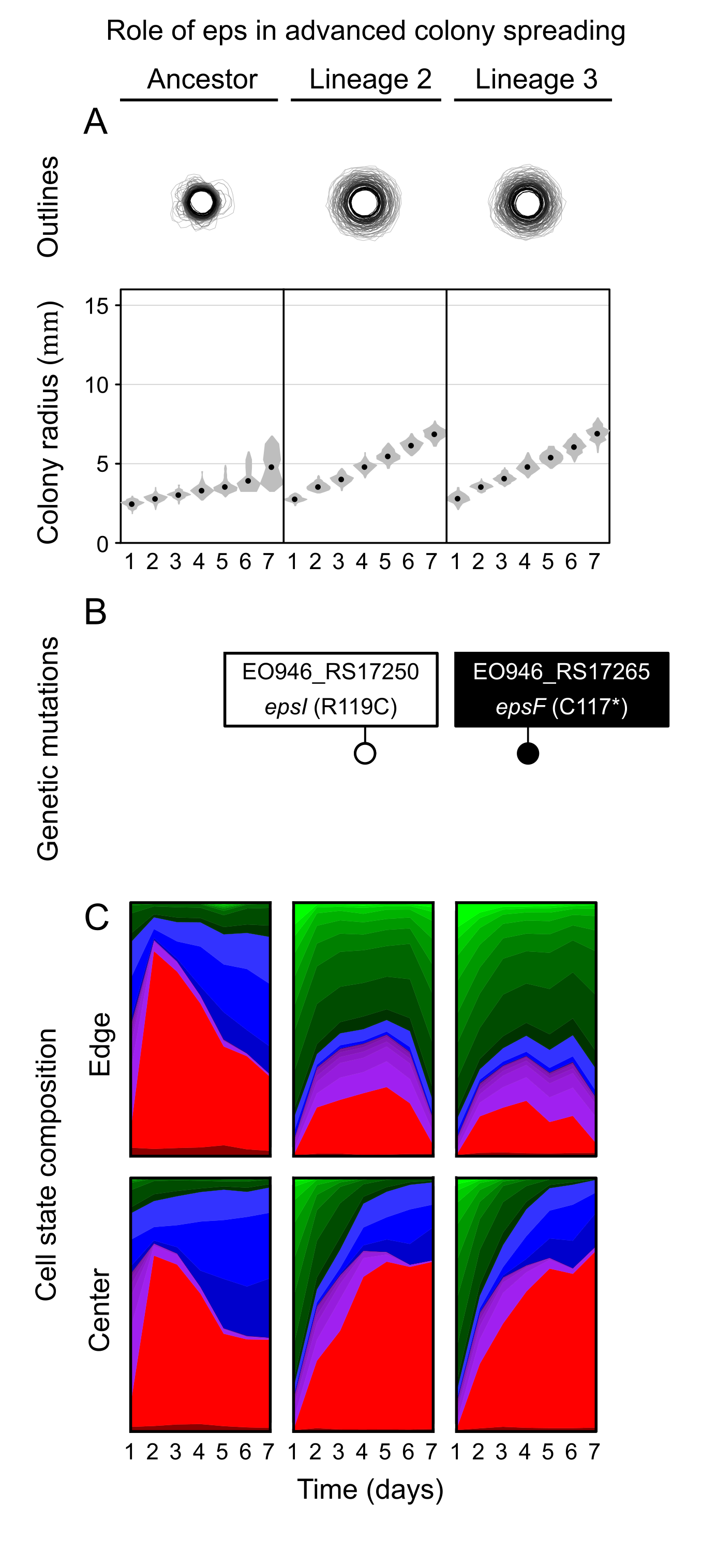

Supplement: S10 Fig — Colony growth in ancestor and eps mutants of lineage 2 and 3. Although lineage 2 and 3 acquired distinct mutations (B) in the eps operon (S3 Table), they show strongly parallel changes in colony expansion (A) and colony composition (C). For figure legend, see caption of Fig 2. Source data can be found in S1 and S2 Data. (TIF) [file pbio.3002338.s010.tif]

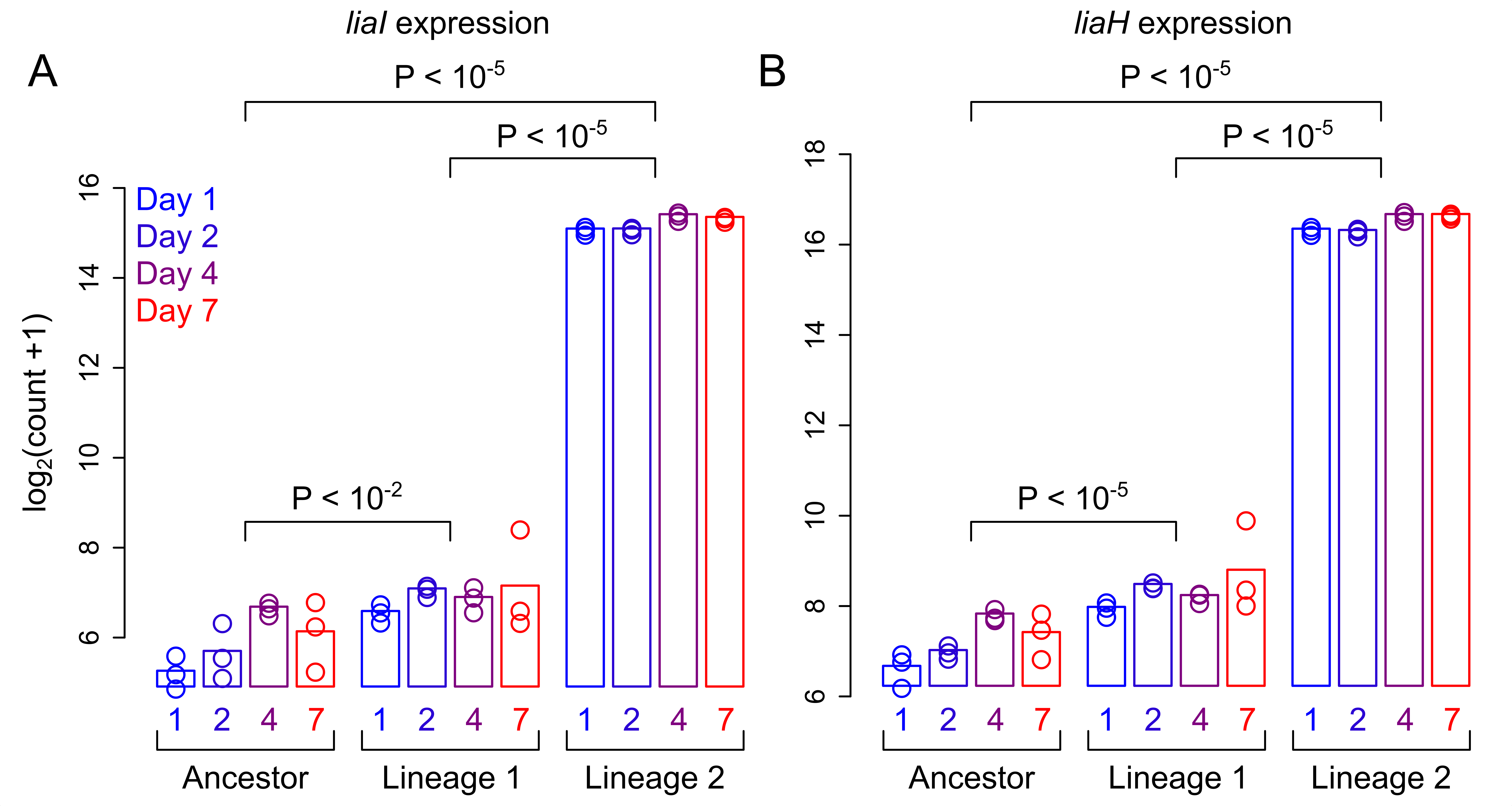

Supplement: S11 Fig — Relative expression (normalized counts) of (A) liaI and (B) liaH in ancestral and evolved population over colony growth cycle: day 1 (blue) to day 7 (red). Bars show average expression (n = 3). Statistics show two-sided Mann Whitney U test, and p-values are adjusted for multiple testing using Benjamini–Hochberg procedure. Source data can be found in S4 and S5 Data. (TIF) [file pbio.3002338.s011.tif]

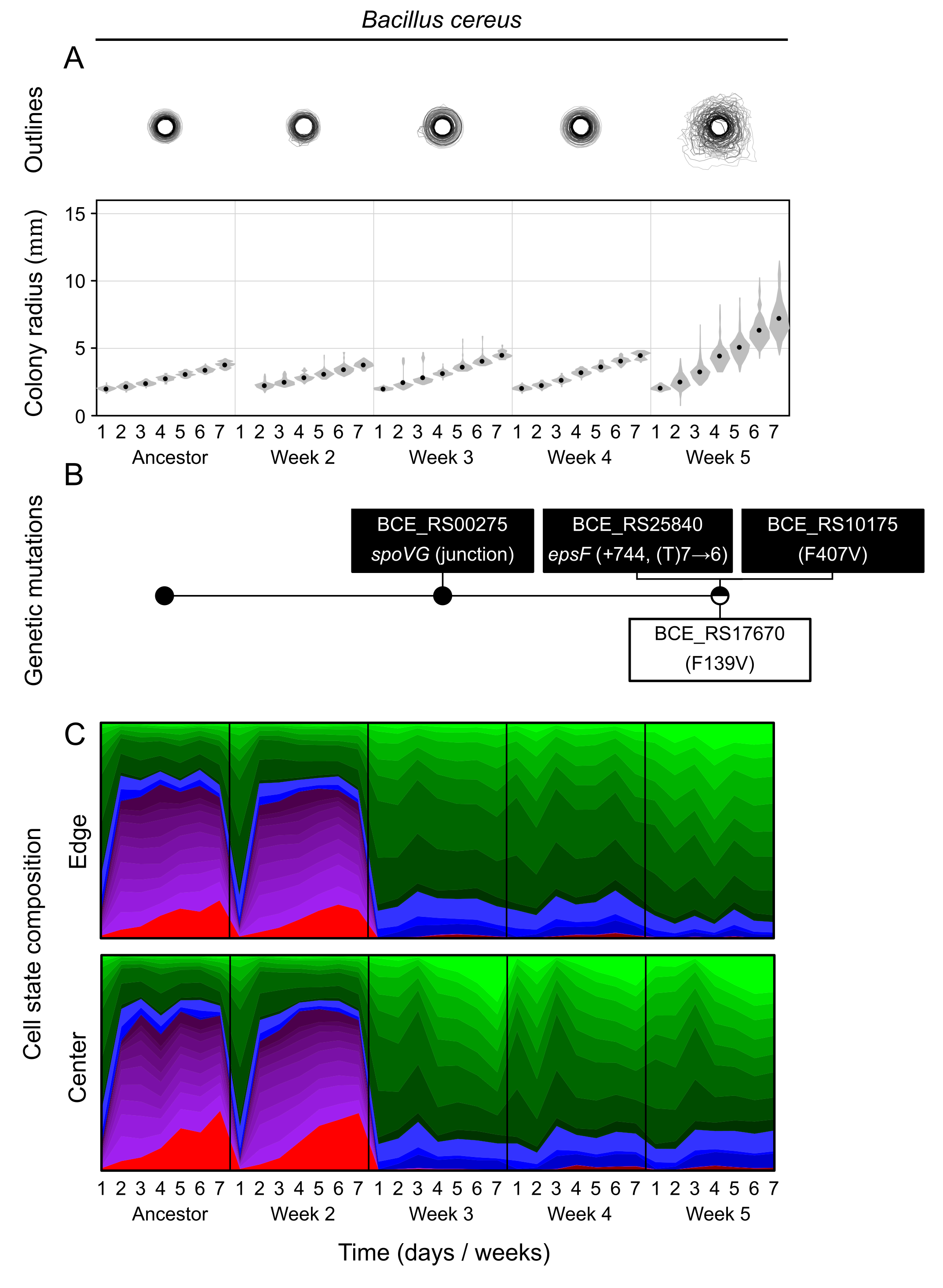

Supplement: S12 Fig — Changes in colony expansion (A), genetic makeup (B), and colony composition (C) during the first weeks of the evolution experiment in B. cereus (see lineage 1 in S4 Table). (A) Grey lines show colony outlines from day 1 (dark grey outline) to day 7 (light grey outline), superimposing different replicate colonies. Graph shows changes in colony radius in time (grey polygon, distribution in colony radius across replicates; black dot, mean; n = 4−14). Source data can be found in S1 Data. (B) Genetic mutations: with mutations that fixed in the population (black) and those that transiently appeared (white). Source data can be found in S3 Data. (C) Colony composition at both colony edge (upper) and center (lower): filamentous cells (green), vegetative cells (blue), sporulating cells (purple), spores (red) (for more details, see legend in Fig 2). Source data can be found in S2 Data. (TIF) [file pbio.3002338.s012.tif]

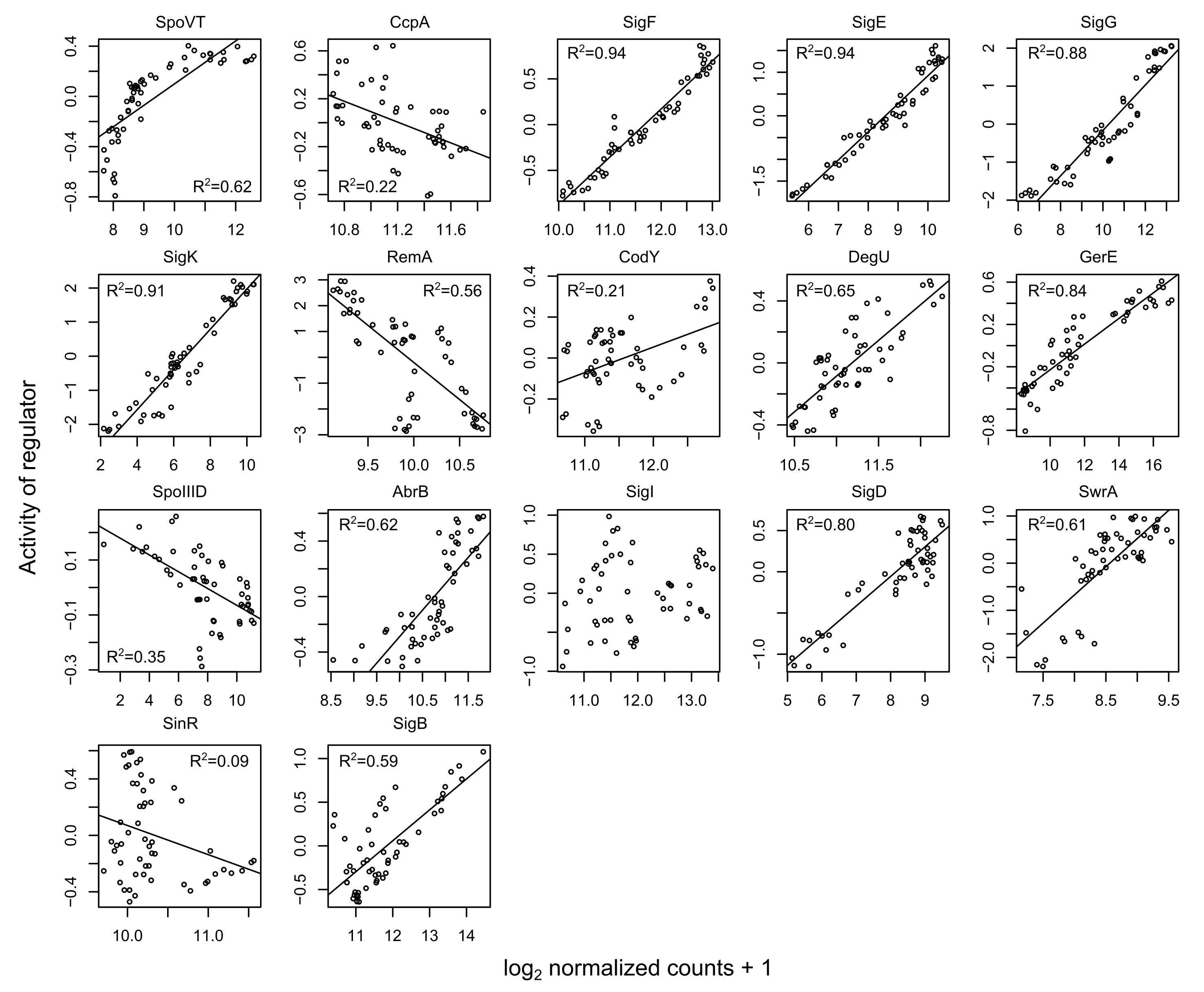

Supplement: S13 Fig — Relation between relative expression and activity for each of the 17 global regulators studied in Fig 4. Lines show linear regression (p<0.05). For some regulators (CcpA, RemA, SpoIIID, and SinR), there is a negative correlation between gene expression and regulatory activity, which could either indicate that there is posttranscriptional feedback that inhibits protein activity upon high expression or that the annotation of regulon is partially incomplete. Source data can be found in S4 and S5 Data. (TIF) [file pbio.3002338.s013.tif]

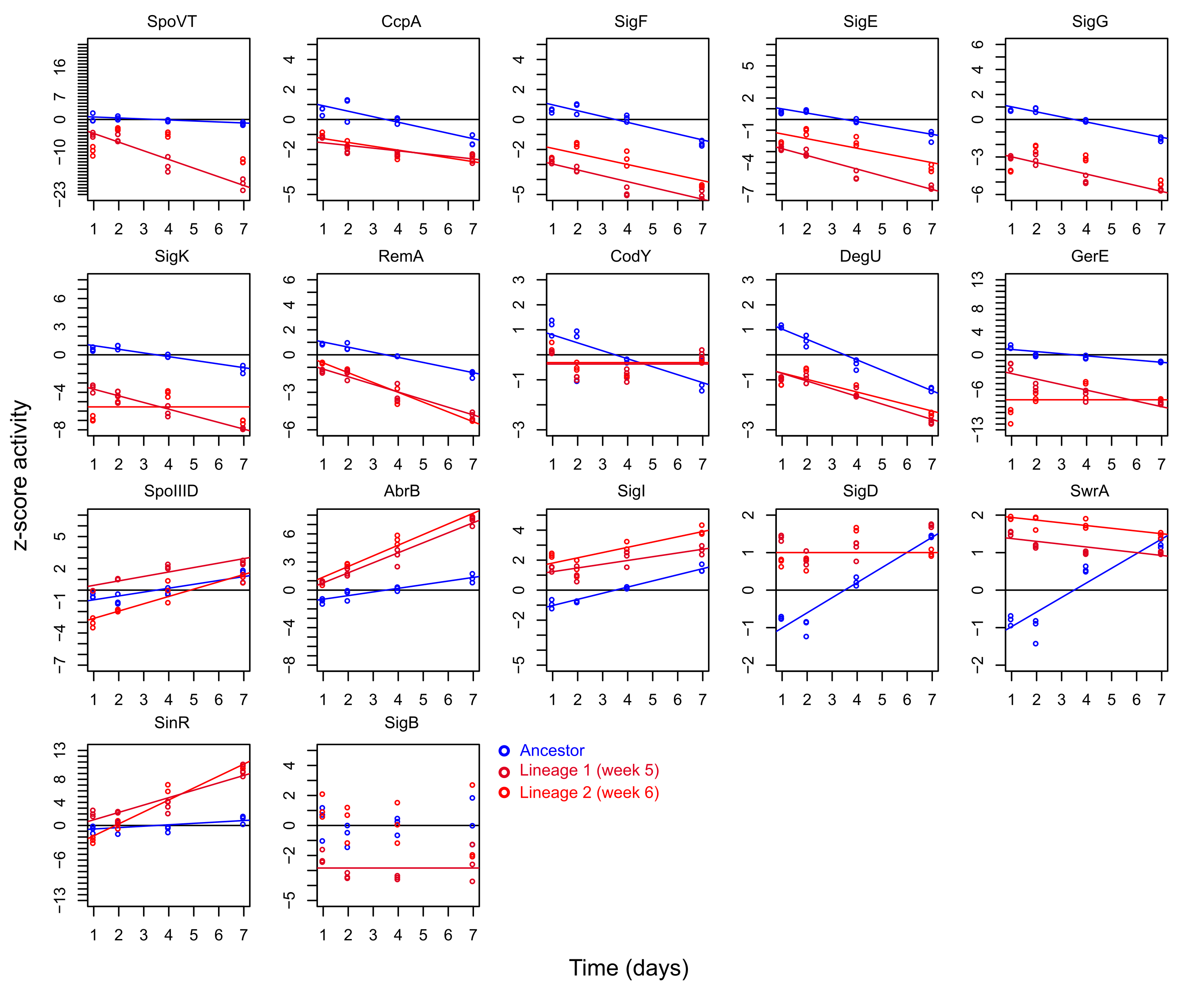

Supplement: S14 Fig — Horizontal black line, reference that indicates no change in activity. Ancestor, blue dots and regression. Evolved population: dark red, lineage 1 and red, lineage 2. Lines show linear regression (p<0.05). Source data can be found in S4 and S5 Data. (TIF) [file pbio.3002338.s014.tif]

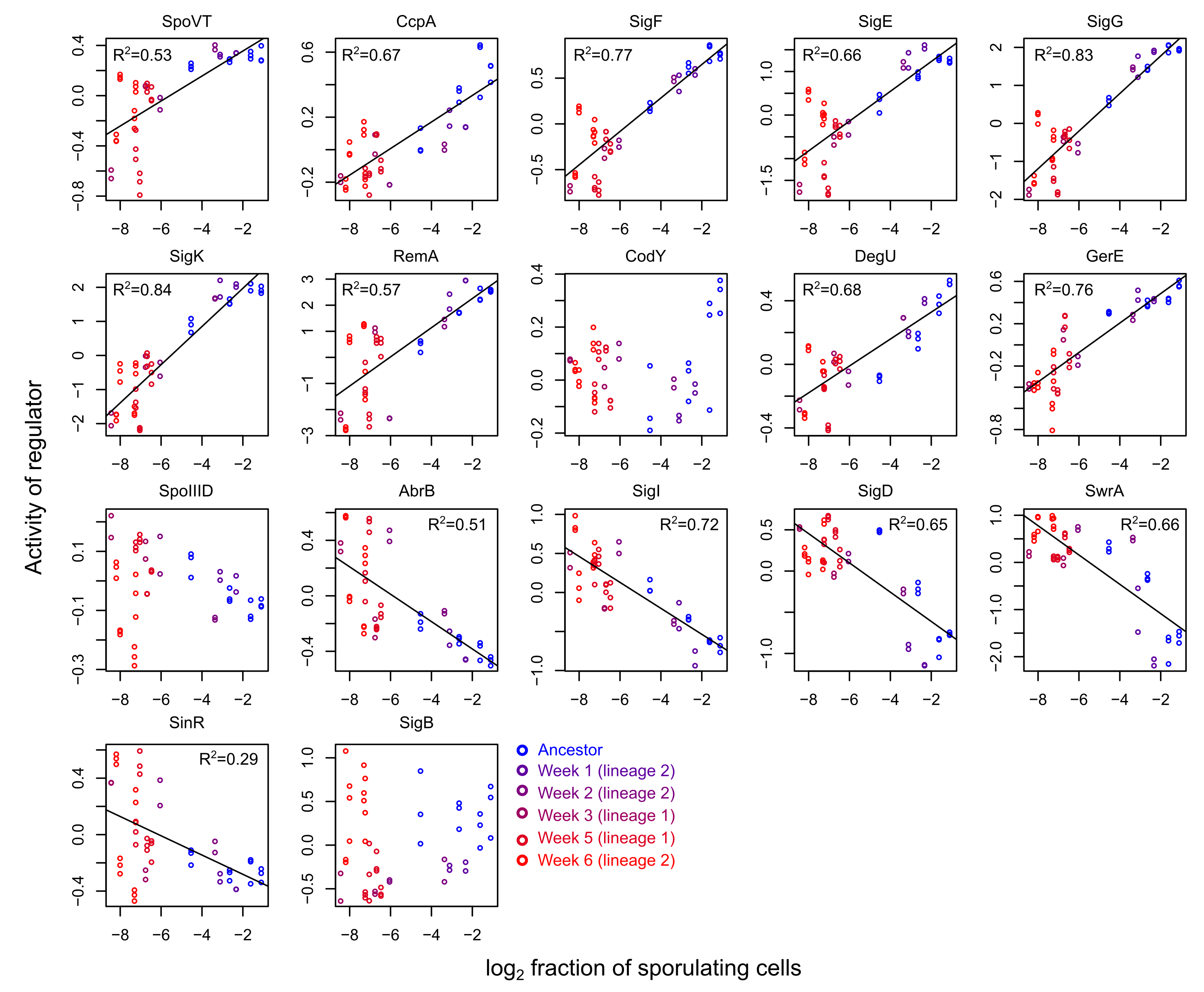

Supplement: S15 Fig — Relation between fraction of sporulating cells and regulatory activity for each of the 17 global regulators in Fig 4: including data from ancestral population (blue) to evolved populations (red). All regulators show strong correlation, except for CodY and SpoIIID. Lines show linear regression (p<0.05). Source data can be found in S4 and S5 Data. (TIF) [file pbio.3002338.s015.tif]

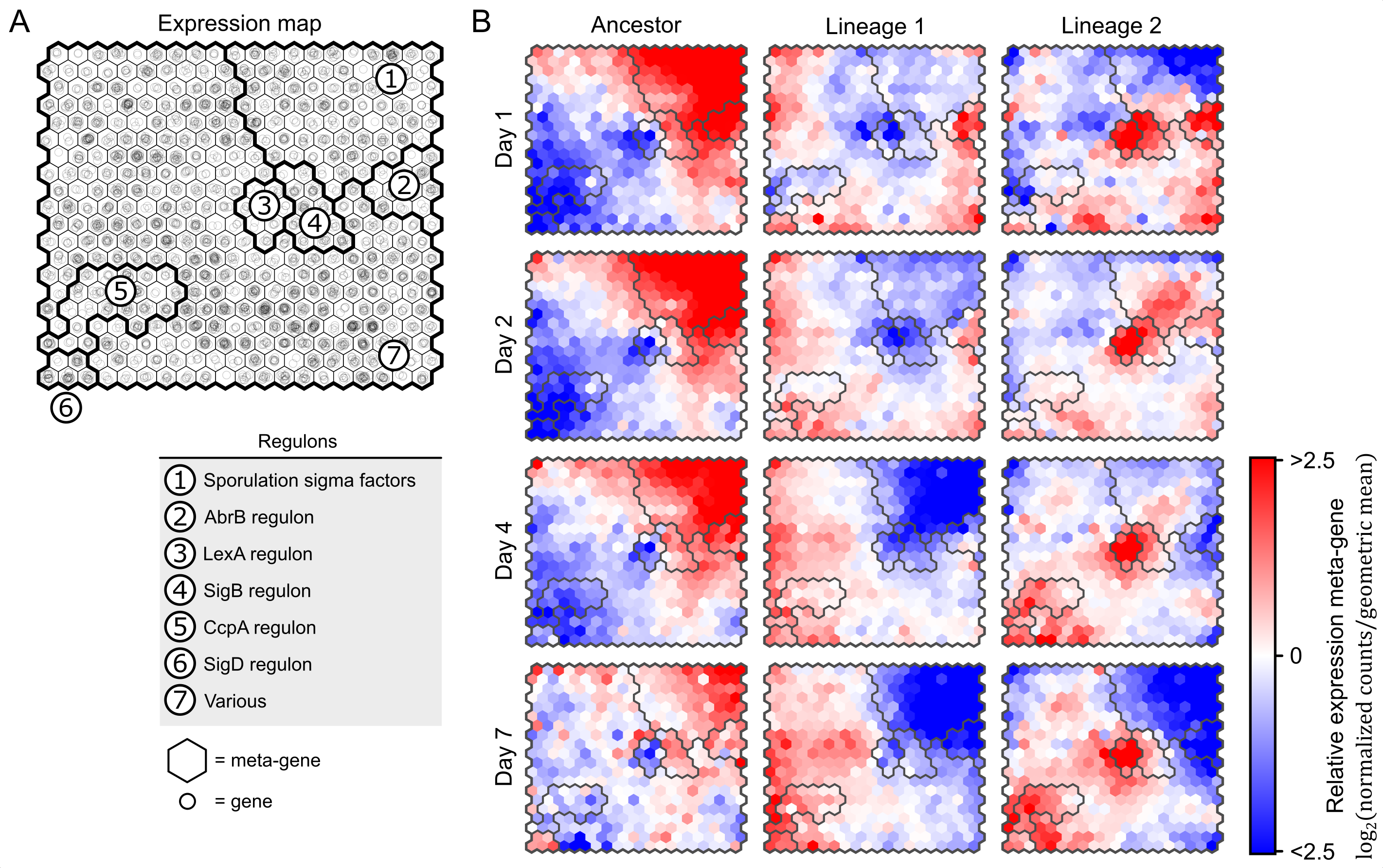

Supplement: S16 Fig — (A) Expression map of genes, based on gene expression profiles in ancestor and evolved colonies, following Kohonen’s self-organizing map. Circles show genes. Hexagons show meta-genes. Genes from the same operon frequently belong to the same meta-gene (S17 Fig). Meta-genes belonging to the same regulon cluster together, as shown for the sporulation sigma factor regulons, AbrB regulon, LexA regulon, SigB regulon, CcpA regulon, SigD regulon. (B) Relative expression of meta-genes at day 1, 2, 4, and 7 of colony growth in ancestral or evolved populations of lineage 1 (week 5) and 2 (week 6): blue, low expression; white, mean expression; red, high expression). Source data can be found in S4 and S5 Data. (TIF) [file pbio.3002338.s016.tif]

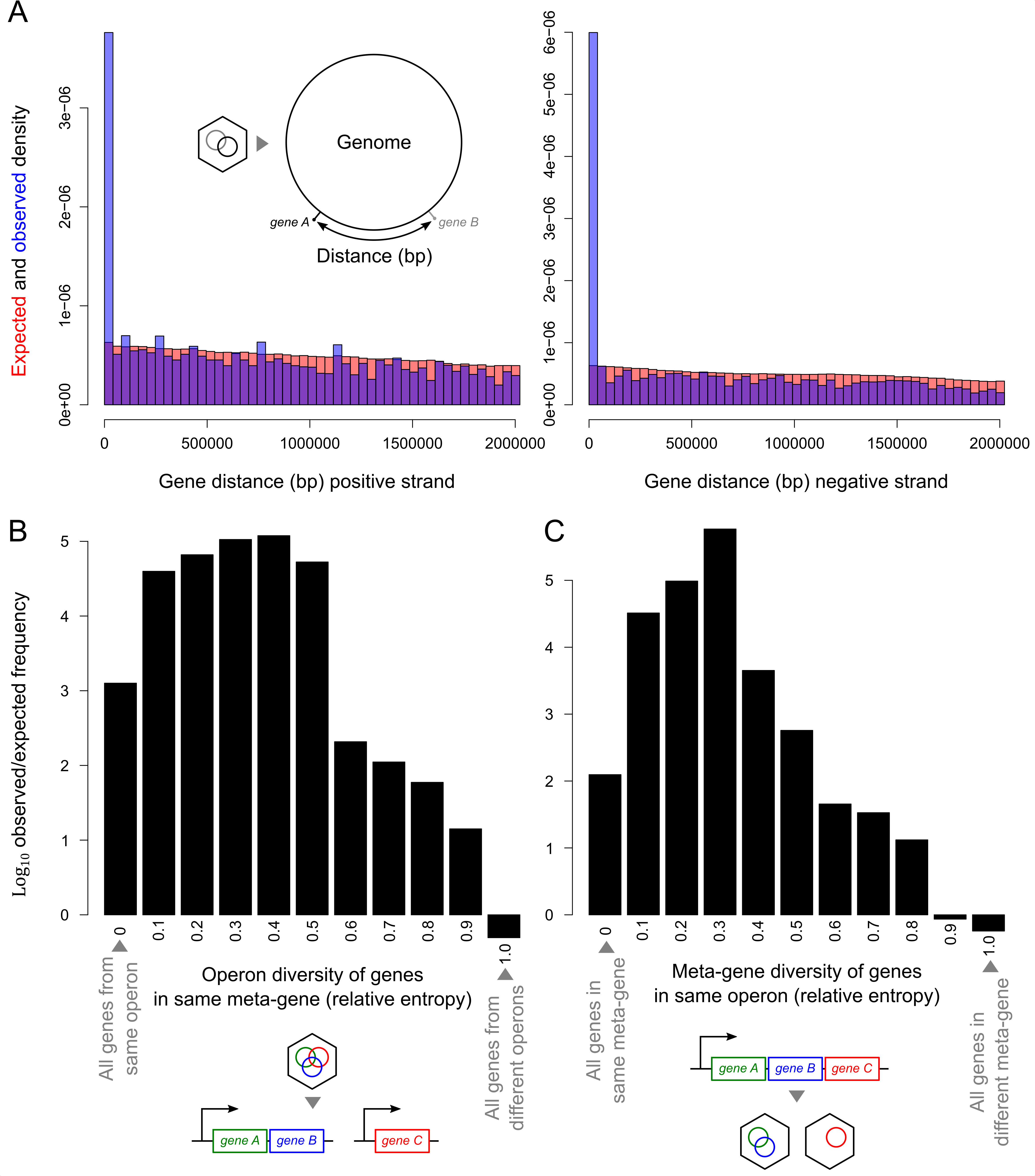

Supplement: S17 Fig — (A) Expected (red) and observed (blue) distance between genes associated with the same meta-gene, calculated by the number of base pairs between their respective start codons on either the positive strand (left) or negative strand (right). (B) Operon diversity among genes belonging to same meta-gene. Operon diversity is calculated using a relative Shannon index: 0 indicates that all genes associated with the same meta-gene also belong to the same operon, and 1 indicates that none of the genes associated with the same meta-gene belong to the same operon. Bars show strong enrichment for low operon diversity, indicating that genes associated with the same meta-gene often belong to the same operon. This enrichment is supported by the results in (A), since—by necessity—genes in the same operon are in close physical proximity on the chromosome. (C) Converse analysis, meta-gene diversity among genes belonging to the same operon. Meta-gene diversity is calculated using a relative Shannon index: 0 indicates that all genes from the same operon are associated with the same meta-gene, and 1 indicates that none of the genes from the same operon are associated with the same meta-gene. Bars show strong enrichment for low meta-gene diversity, indicating that genes from the same operon are often associated with same meta-gene. This enrichment logically follows from the results in (B). Source data can be found in S4 and S5 Data. (TIF) [file pbio.3002338.s017.tif]

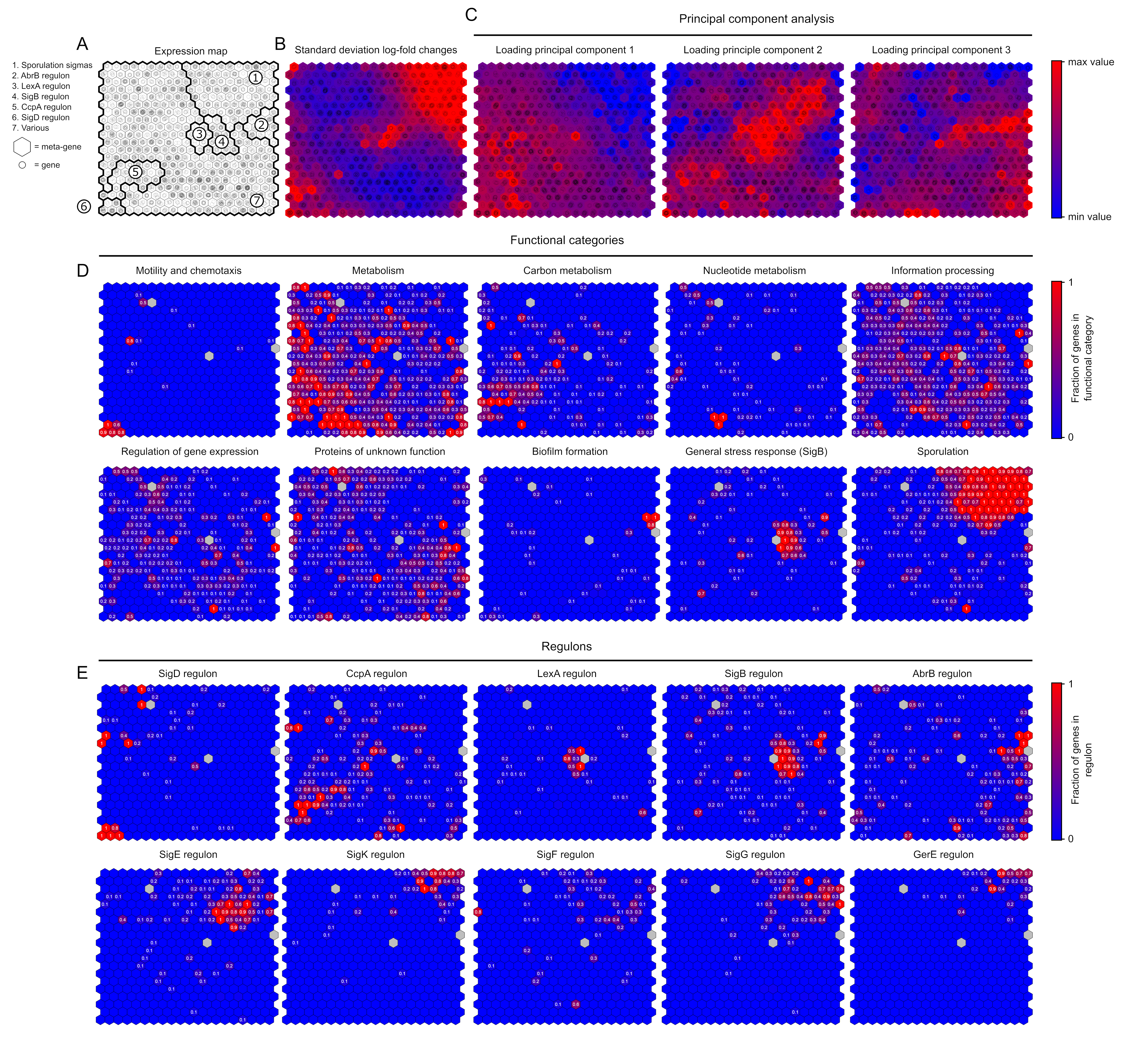

Supplement: S18 Fig — (A) Overview of expression map as shown in S16A Fig. (B) Standard deviation in meta-gene expression across expression profiles (i.e., log-fold changes): from low (blue) to high (red) variability in expression. (C) Loading of meta-genes on principal component 1, 2, and 3: from low loading (blue) to high loading (red). (D) Fraction of genes within meta-gene belonging to functional category as defined by the SubtiWiki database [74–76]: motility and chemotaxis, metabolism, carbon metabolism, nucleotide metabolism, information processing, regulation of gene expression, proteins of unknown function, biofilm formation, general stress response, and sporulation. (E) Fraction of genes within meta-gene belonging to a particular regulon as defined by the SubtiWiki database [74–76]: SigD regulon, CcpA regulon, LexA regulon, SigB regulon, AbrB regulon, SigE regulon, SigK regulon, SigF regulon, SigG regulon, and GerE regulon. In (D) and (E), blue indicates that none of the genes associated with meta-gene belong to functional category/regulon; red indicates that all genes belong to functional category/regulon. White number indicates fraction of genes belonging to functional category/regulon. Source data can be found in S4 and S5 Data. (TIF) [file pbio.3002338.s018.tif]

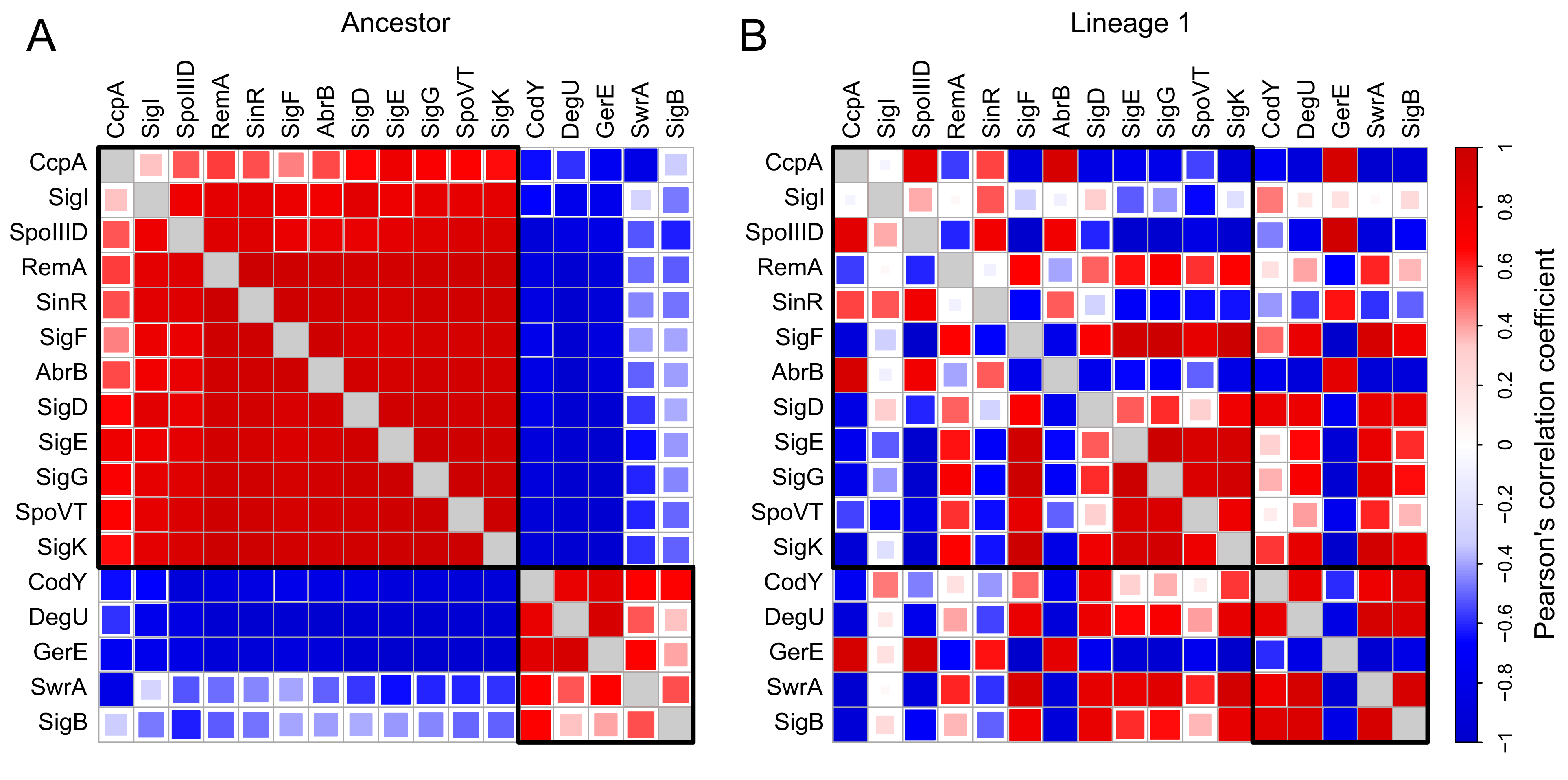

Supplement: S19 Fig — Color and size of squares show Pearson’s correlation coefficient between activity of regulators in ancestral population (A) and evolved population (B). Coactivation pattern is strongly disrupted in evolved population relative to that observed in the ancestor. Source data can be found in S4 and S5 Data. (TIF) [file pbio.3002338.s019.tif]

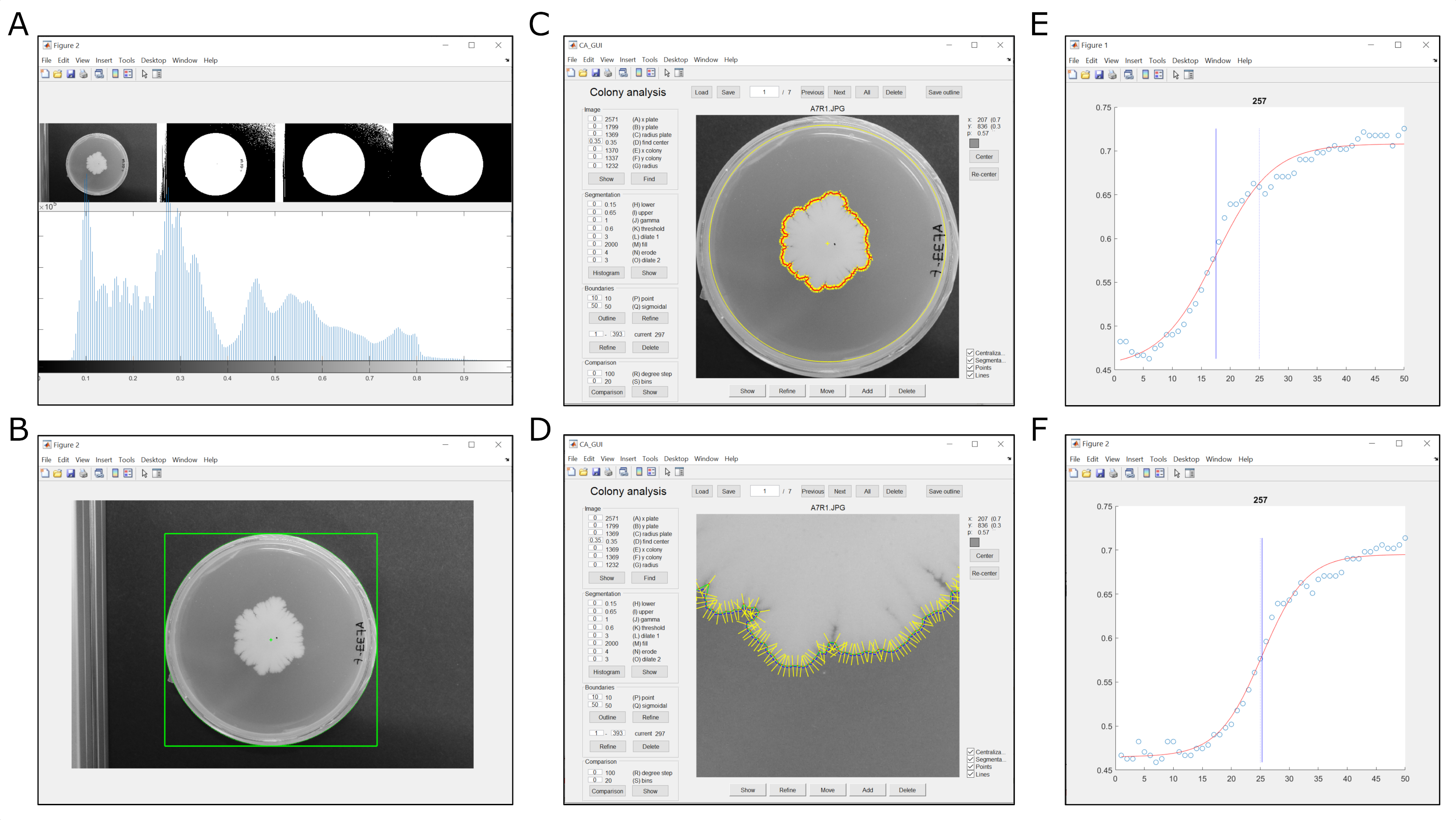

Supplement: S20 Fig — We designed a custom-made image analysis software in Matlab to determine the colony outlines in 1,388 colony images. The general user interface is shown in (C). First, the software automatically detects the Petri dish, using a simple segmentation procedure (B). Segmentation occurs through a few simple steps, which associated parameter settings can be optimized, by displaying the segmentation procedure in (A). After detecting the Petri dish (B), the colony outline is automatically detected (C) using another simple segmentation procedure, whose parameters could be optimized. To refine the colony boundary, small perpendicular lines are drawn along the colony outline; see yellow lines in (C) and (D), along which the pixel intensity values are measured. These values are expected to fall along a sigmoidal curve (red lines in (E) and (F)). In the refinement step, the outline (vertical blue solid line in (E)) is optimized such that it falls at the inflection point of the sigmodal curve (vertical blue dotted line in (F)). Panels (E) and (F) show how the boundary is refined at a single point along the colony outline. This procedure is done for the entire outline. The refinement step assures that the colony outline is perfectly placed, despite local differences in light intensities (i.e., due to shading or otherwise). For the rare cases where automatic refinement fails, one can also manually adjust the outline, by zooming in (as shown in (D)), and then removing, moving, or adding points along the outline. For a full description of the image analysis software as well as the Matlab script, see our Github repository https://github.com/jordivangestel/PLoS-Biology-2023. (TIF) [file pbio.3002338.s020.tif]
