# Supplementary material for: Pleiotropic hubs drive bacterial surface competition through parallel changes in colony composition and expansion
Source: PLoS Biol. 2023 Oct 16;21(10):e3002338. doi: 10.1371/journal.pbio.3002338 (PMC10578586; doi:10.1371/journal.pbio.3002338)
Supplement: S1 Text — (PDF) [file pbio.3002338.s021.pdf]

S1 Text. Model on surface growth

To investigate what colony properties are favored during surface colonization, we constructed a minimal phenomenological model to emulate *Bacilli* colony growth. We assume cells grow on a flat nutritious substrate, where they can grow and form a colony. Cells consume nutrients, divide, and – when nutrients get depleted – sporulate. For simplicity, we assume colonies are radially symmetrical, which allows us to simulate colony growth along its one-dimensional radius, thereby ignoring the complexities of two-dimensional colony expansion (as modeled before[1,2]). We monitor the nutrient concentration (N), cell density (C) and spore density (S). We assume that cells grow at a rate (μ) that is proportional to the nutrient concentration (N). As cells divide, the local cell density increases. Following experimental evidence[3], we assume that cells have a fixed probability to sporulate (p) when the nutrient concentration drops below a certain pre-determined threshold (T_N). Spores are dormant and consume no nutrients. Based on these assumptions, colony growth can be simulated by the following equations:

$$[1] \quad \frac{\partial N(r)}{\partial t} = D_N \nabla^2 N(r) - \frac{\mu}{Y} \cdot C(r) \cdot N(r)$$

$$[2] \quad \begin{cases} if\ N(r) \geq T_N, & \frac{\partial C(r)}{\partial t} = D_B \nabla^2 C(r) + \mu \cdot C(r) \cdot N(r) - p \cdot N(x) \\ if\ N(r) < T_N, & \frac{\partial C(r)}{\partial t} = D_B \nabla^2 C(r) + \mu \cdot C(r) \cdot N(r) - p \cdot C(r) \end{cases}$$

$$[3] \quad \begin{cases} if\ N(r) \geq T_N, & \frac{\partial S(r)}{\partial t} = D_B \nabla^2 S(r) - p \cdot N(x) \\ if\ N(r) < T_N, & \frac{\partial S(r)}{\partial t} = D_B \nabla^2 S(r) + p \cdot C(r) \end{cases}$$

$$[4] \quad B(r) = C(r) + S(r)$$

$N(r)$ is the nutrient concentration at radius, r, from the colony center. $C(r)$ is the cell density at radius, r. $S(r)$ is the spore density at radius, r. $B(r)$ is the total biomass at radius, r, which we define as the sum of spores and cells. The nutrient concentration is scaled between 0 and 1, such that the highest nutrient concentration equals 1. Nutrients are consumed at a constant rate, μ/Y , where each unit of nutrients yields Y cells. Nutrients furthermore have a constant diffusion rate, D_N. As described above, we assume cells consume nutrients at a constant rate (μ) and when nutrients become sparse ($N < T_N$) cells have a fixed probability, p, to develop into spores. For simplicity, we model colony expansion using a biomass diffusion term, D_B, where $D_B \ll D_N$. The biomass diffusion coefficient (D_B) determines the relative degree of lateral colony expansion as opposed to vertical

colony growth: when D_B is low, colonies are small and thick; when D_B is high, colonies are large and flat. We examine colony growth in a regular sized Petri dish, where $r \in (0, R)$ and $R = 42\ mm$. Following our experiments, we monitor colony growth for one week, $t \in (0, T)$, where $T = 7 \cdot 24 \cdot 60 \cdot 60 = \sim 6 \cdot 10^5\ s$. We furthermore assume that at the onset of colony growth a low density of cells occurs within the inoculation radius, $r_{init} = 2mm$, resulting in the following starting conditions ($t = 0$): $N(r) = 1,\ C(r < r_{init}) =\ 10^{-2};\ C(r > r_{init}) = 0;\ S(x) = 0$.

For Figure S1A, we numerically solve the above set of ordinary differential equations using the following parameter settings:

Description	Parameter	Figure 1
Nutrient diffusion coefficient	$D_N(mm^2 s^{-1})$	$10^{-4}\ mm^2 s^{-1}$
Biomass diffusion coefficient	$D_B(mm^2 s^{-1})$	$10^{-7}\ mm^2 s^{-1}$
Growth rate	$\mu(h^{-1})$	$0.5\ h^{-1}$
Nutrient threshold	T_N	0.2
Yield	Y	10
Sporulation rate	$p(h^{-1})$	$0.1\ h^{-1}$

These parameter settings are obtained after scaling to the maximum nutrient concentration to 1. The bacterial density is thus expressed relative to the nutrient concentration, i.e., density per unit of nutrients. The parameters are in part based on experimental data, such as *B. subtilis'* division rate ($\sim 0.5\ h^{-1}$) and colony size (diffusion coefficient of $10^{-7}\ mm^2 s^{-1}$ leads to a colony size that approximates that of the ancestor in Figure 1C) as well as ballpark estimates.

To explore how surface competition affects selection, we performed pairwise competition simulations between mutant and wildtype genotypes. Where mutant genotypes are assumed to have alternative biomass diffusion (D_B), sporulation (p) or growth (μ) rates than the wildtype. The mutant and wildtype are assumed to be homogeneously distributed in the inoculum at equal abundance and can subsequently grow for 7 days. Following our experiment, where we scrape cells from the outermost colony edge using an inoculation loop, we determine competitive success by measuring the fraction of mutant biomass at the edge of the colony, in a zone of $2.5mm$ from the outermost colony edge (which equals the radius of an inoculation loop). The mutant is favored by selection when it is more abundant at the colony edge than the wildtype. Figure S1B shows the outcome of competition.

The model makes two key predictions. First, spatial competition favors mutants with higher expansion rates (D_B), lower sporulation probabilities (p), and higher division rates (μ) (S1B Figure). Second, depending on the parameter that evolves, selection has a different impact on colony size and composition: higher D_B values strongly affect both colony size and composition; lower p values mostly affect colony composition; and higher μ values have nearly no effect on either colony size or

composition. These predictions make intuitive sense: faster expansion rates allow cells to escape nutrient depletion, and therefore lead to a lower fraction of spores, while reduced sporulation rates are effectively increase the pool of dividing cells, which have a minimal effect on colony expansion in our model.

References

1. van Gestel, J., Weissing, F. J., Kuipers, O. P. & Kovács, Á. T. Density of founder cells affects spatial pattern formation and cooperation in *Bacillus subtilis* biofilms. *ISME J* **8**, 2069–2079 (2014).
2. Lee, H., Gore, J. & Korolev, K. S. Slow expanders invade by forming dented fronts in microbial colonies. *PNAS* **119**, e2108653119 (2022).
3. Russell, J. R., Cabeen, M. T., Wiggins, P. A., Paulsson, J. & Losick, R. Noise in a phosphorelay drives stochastic entry into sporulation in *Bacillus subtilis*. *EMBO J* **36**, 2856–2869 (2017).
