# Supplementary material for: Pleiotropic hubs drive bacterial surface competition through parallel changes in colony composition and expansion
Source: PLoS Biol. 2023 Oct 16;21(10):e3002338. doi: 10.1371/journal.pbio.3002338 (PMC10578586; doi:10.1371/journal.pbio.3002338)
Supplement: S2 Text — (PDF) [file pbio.3002338.s022.pdf]

S2 Text. Extended evaluation of mutations in lineage 1 and 2

2.1 Lineage 1. Loss-of-function mutation in *ricT*

The *ricT* mutation in lineage 1 resulted in a premature stop codon (S3 Table), several lines of evidence suggest that this mutation resulted in a loss of function. First, it was previously shown that *ricT* null mutants affect the expression of other genes in the Y-complex. Our results recapitulate these findings (S5 Figure): *ricF* expression increases ($log_2FC = 1.2, P < 10^{-7}$) and *ricA* expression decreases ($log_2FC = -1.7, P < 10^{-16}$)[1]. Second, like observed before, we found that our *ricT* mutant resulted in a loss of Y-complex dependent cleavage sites in polycistronic mRNA, like the cleavage of *cggR-gapA* mRNA[2,3] (S6 Figure; Commichau et al. 2009; DeLoughery et al. 2016; 2018). Third, the Y-complex is also involved in the turnover of S-box riboswitches[1]. Accordingly, we found that the *ricT* mutant is enriched for S-box riboswitches among the set of differentially expressed genes ($P < 0.01$; see Methods in S5 Text). Altogether, these results corroborate that the mutation in *ricT* represents a knockout mutation.

2.2 Lineage 2. Reduced expression of *rny*

As described in the main text, the mutation upstream of *rny* in lineage 2 occurs in the -10 element of the *rny* promoter (from A_{-8} to T_{-8}, S7 Figure)[4,5] and resulted in an 8-fold lower *rny* expression ($log_2FC = -3.0, P < 10^{-16}$) (S5 Figure). For comparison, *rny* expression was unchanged in the *ricT* mutant of lineage 1 (S5 Figure). Another gene in the same operon, *ymdB*, also showed strongly reduced expression as a consequence of the mutation in the *rny* promoter ($log_2FC = -2.1, P < 10^{-16}$). Like a *rny* null mutant, null mutants in *ymdB* were previously shown to also reduce EPS production[6,7], which suggests that the pleiotropic effects of the *rny* mutant might in part result as well from the reduced expression of *ymdB*.

2.3 Lineage 2. Loss-of-function mutation of *lexA*

LexA is the transcriptional repressor of the SOS response, a widely observed stress response that is normally triggered in response to DNA damage[8,9]. In lineage 2, we observed a mutation in *lexA* that results in an amino acid substitution close to the DNA binding site, likely rendering it non-functional (S8A Figure)[10,11]. This is supported by our gene expression data: Figure S8B shows that the *lexA* mutant results in the constitutive expression of the SOS response, which includes the expression of *yneA*[12,13] ($log_2FC = 7.6, P < 10^{-16}$), i.e., a gene underlying SOS filamentation. In addition, LexA is also known to suppress its own expression. Accordingly, we observed that our *lexA* mutation results in an increased expression of *lexA* ($log_2FC = 1.9, P < 10^{-14}$). Consistent with previous studies, we also observe that our *lexA* mutant reduces the fraction of sporulating cells (see S4 Figure). However, unlike

previous studies, which suggests sporulation is suppressed through the Sda-dependent inhibition of Spo0A activity[14,15], we do not find any changes in *sda* expression[14,15]. The SOS response might thus affect sporulation through an alternative mechanism. Finally, in week 7 of lineage 2, we observed a maladaptive mutation targeting the essential LysM peptidoglycan-binding domain of YneA[13,16], which strongly reduces filamentation and results in smaller colonies (S9 Figure). Since YneA mediates SOS filamentation, the *yneA* mutant confirms that SOS filamentation is responsible for the advanced colony spreading of the *lexA* mutant that is observed in week 6 of the evolution experiment (Figure 2). Since the mutation in *yneA* abolishes filamentation, and results in smaller colonies, it is maladaptive and does not fix in the population (S3 Table).

2.4 Description of other mutations in lineage 1 and 2

Besides the mutations affecting the global regulators discussed above, in both lineage 1 and 2, we found various additional mutations. In lineage 1, there were only few additional mutations (Figure 2C): two synonymous substitutions in *pnp* and EO946_RS10365 respectively, without any visible effect on either colony growth or composition, and one frameshift mutation in *epsK* that led to strongly increased colony expansion. EpsK is necessary for the transport of extracellular poly-N-acetylglucosamine[17]. The frameshift mutation therefore likely prevents EPS production, which promotes colony expansion under our growth conditions.

In lineage 2 we found a more intricate series of mutations (Figure 2D). In week 1, we observed mutations in both *epsI* and *polX* (besides a synonymous mutation in EO946_RS19755). *PolX* encodes DNA polymerase X, which is involved in DNA repair. Both in terms of colony growth and composition, the double *epsI*, *polX* mutant was indistinguishable from a null mutant in EPS production (as observed in lineage 3 of *B. subtilis*; S10 Figure), suggesting that the *epsI* mutant in week 1 underlies the increase in colony size. None of the mutations in week 1 of the experiment fix in the population.

In week 2, we observed a mutation upstream of *qdoI* (S7 Figure), which encodes a dioxygenase responsible for degrading a plant-derived flavonoid called quercetin[18,19]. This mutation occurs in the Shine-Dalgarno sequence, and is therefore expected to lower the translation efficiency of *qdoI* mRNA. The *qdoI* mutation occurs in the same week as the *rny* mutant and since the observed phenotypic effects match closely to those reported in previous studies on *rny* depletion[20,21], we suspect that the *qdoI* mutant has no or minor phenotypic effects only.

In week 3 and 5, we observed an in-frame deletion in *liaF*, which had minimal effect on colony growth and composition (Figure 2B,D,F). The *liaF* mutation appeared first in week 3 and fixed in week 5. *liaF* encodes the membrane-bound protein LiaF, which inhibits the LiaRS-mediated cell-envelope stress response[22,23], thereby enhancing stress resistance[23,24]. The stress response includes the *liaIH* operon,

accordingly we find a strong increase *liaIH* expression (S11 Figure). In fact, *liaI* and *liaH* showed the largest increase in expression recorded among our expression profiles (*liaI*: $log_2FC = 9.6, P < 10^{-16}$; *liaH*: $log_2FC = 9.5, P < 10^{-16}$). Since the *liaF* mutant has minimal effect on colony growth and composition, we speculate that it might positively affect the cell division rate – which according to our model (Figure 1) – hardly affects colony-level phenotypes.

In week 4, we also find two non-synonymous mutations, in *nhaX* and *rplI*. Both these mutations fail to fixate and have no apparent effect on colony growth or composition.

References

1. DeLoughery, A., Lalanne, J. B., Losick, R. & Li, G. W. Maturation of polycistronic mRNAs by the endoribonuclease RNase Y and its associated Y-complex in *Bacillus subtilis*. *PNAS* **115**, E5585–E5594 (2018).
2. Commichau, F. M. *et al.* Novel activities of glycolytic enzymes in *Bacillus subtilis*: interactions with essential proteins involved in mRNA processing. *Mol Cell Proteomics* **8**, 1350–1360 (2009).
3. DeLoughery, A., Dengler, V., Chai, Y. & Losick, R. Biofilm formation by *Bacillus subtilis* requires an endoribonuclease-containing multisubunit complex that controls mRNA levels for the matrix gene repressor SinR. *Mol Microbiol* **99**, 425–437 (2016).
4. Murakami, K. S., Masuda, S., Campbell, E. A., Muzzin, O. & Darst, S. A. Structural basis of transcription initiation: an RNA polymerase holoenzyme-DNA complex. *Science* **296**, 1285–1290 (2002).
5. Feklistov, A. & Darst, S. A. Structural basis for promoter −10 element recognition by the bacterial RNA polymerase σ subunit. *Cell* **147**, 1257–1269 (2011).
6. Diethmaier, C. *et al.* The YmdB phosphodiesterase is a global regulator of late adaptive responses in *Bacillus subtilis*. *J Bacteriol* **196**, 265–275 (2014).
7. Kampf, J. *et al.* Selective pressure for biofilm formation in *Bacillus subtilis*: differential effect of mutations in the master regulator SinR on bistability. *mBio* **9**, e01464-18 (2018).
8. Haijema, B. J. *et al.* Regulated expression of the *dinR* and *recA* genes during competence development and SOS induction in *Bacillus subtilis*. *Mol Microbiol* **22**, 75–85 (1996).
9. Au, N. *et al.* Genetic composition of the *Bacillus subtilis* SOS system. *J Bacteriol* **187**, 7655–7666 (2005).
10. Mazón, G., Campoy, S., Erill, I. & Barbé, J. Identification of the *Acidobacterium capsulatum* LexA box reveals a lateral acquisition of the Alphaproteobacteria *lexA* gene. *Microbiology* **152**, 1109–1118 (2006).

11. Zhang, A. P. P., Pigli, Y. Z. & Rice, P. A. Structure of the LexA-DNA complex and implications for SOS box measurement. *Nature* **466**, 883–886 (2010).
12. Kawai, Y., Moriya, S. & Ogasawara, N. Identification of a protein, YneA, responsible for cell division suppression during the SOS response in *Bacillus subtilis*. *Mol Microbiol* **47**, 1113–1122 (2003).
13. Mo, A. H. & Burkholder, W. F. YneA, an SOS-induced inhibitor of cell division in *Bacillus subtilis*, is regulated posttranslationally and requires the transmembrane region for activity. *J Bacteriol* **192**, 3159–3173 (2010).
14. Burkholder, W. F., Kurtser, I. & Grossman, A. D. Replication initiation proteins regulate a developmental checkpoint in *Bacillus subtilis*. *Cell* **104**, 269–279 (2001).
15. Lenhart, J. S., Schroeder, J. W., Walsh, B. W. & Simmons, L. A. DNA repair and genome maintenance in *Bacillus subtilis*. *Microbiol Mol Biol Rev* **76**, 530–564 (2012).
16. Mesnage, S. *et al.* Molecular basis for bacterial peptidoglycan recognition by LysM domains. *Nat Commun* **5**, 4269 (2014).
17. Marvasi, M., Visscher, P. T. & Martinez, L. C. Exopolymeric substances (EPS) from Bacillus subtilis: polymers and genes encoding their synthesis. *FEMS Microbiology Letters* **313**, 1–9 (2010).
18. Bowater, L., Fairhurst, S. A., Just, V. J. & Bornemann, S. *Bacillus subtilis* YxaG is a novel Fe-containing quercetin 2,3-dioxygenase. *FEBS Letters* **557**, 45–48 (2004).
19. Hirooka, K. & Fujita, Y. Excess production of *Bacillus subtilis* quercetin 2,3-dioxygenase affects cell viability in the presence of quercetin. *Biosci Biotechnol Biochem* **74**, 1030–1038 (2010).
20. Figaro, S. *et al. Bacillus subtilis* mutants with knockouts of the genes encoding ribonucleases RNase Y and RNase J1 are viable, with major defects in cell morphology, sporulation, and competence. *J Bacteriol* **195**, 2340–2348 (2013).
21. Koo, B. M. *et al.* Construction and analysis of two genome-scale deletion libraries for *Bacillus subtilis*. *Cell Syst* **4**, 291-305.e7 (2017).
22. Mascher, T., Margulis, N. G., Wang, T., Ye, R. W. & Helmann, J. D. Cell wall stress responses in *Bacillus subtilis*: the regulatory network of the bacitracin stimulon. *Mol Microbiol* **50**, 1591–1604 (2003).
23. Jordan, S., Junker, A., Helmann, J. D. & Mascher, T. Regulation of LiaRS-dependent gene expression in *Bacillus subtilis*: identification of inhibitor proteins, regulator binding sites, and target genes of a conserved cell envelope stress-sensing two-component system. *J Bacteriol* **188**, 5153–5166 (2006).
24. Radeck, J., Fritz, G. & Mascher, T. The cell envelope stress response of *Bacillus subtilis*: from static signaling devices to dynamic regulatory network. *Curr Genet* **63**, 79–90 (2017).
