# Supplementary material for: Pleiotropic hubs drive bacterial surface competition through parallel changes in colony composition and expansion
Source: PLoS Biol. 2023 Oct 16;21(10):e3002338. doi: 10.1371/journal.pbio.3002338 (PMC10578586; doi:10.1371/journal.pbio.3002338)
Supplement: S3 Text — (PDF) [file pbio.3002338.s023.pdf]

S3 Text. Expression maps

In the main text we examine temporal expression changes by focusing on changes in regulatory activities. For this method, we rely on predefined regulons, as provided by the *Subti*Wiki database[1–3], to determine how regulators change activity in time. To complement this approach, we also examined gene expression changes in lineage 1 and 2 directly by applying an unsupervised machine-learning approach, called Kohonen's self-organizing maps[4,5]. With this approach, we can study how genes and regulons change expression in time without the need to predefine such regulons in advance. In short, in a Kohonen's self-organizing map, genes are projected on a two-dimensional hexagonal grid according to the similarity of their expression across samples: genes with similar expression profiles map closely, or even inside the same grid element, and genes with distinct expression profiles map far apart.

Figure S16A shows the resulting expression map. Each circle represents an individual gene and each hexagon can be viewed as a meta-gene: a cluster of genes with near-identical expression. Genes that belong to the same meta-gene are often part of the same operon and are thus transcribed from the same promoter (S17 Figure). Genes in neighboring meta-genes have a similar expression as well, for example because they belong to the same regulon[6]. In this way, the expression map visualizes distinct layers of regulation (e.g., operon and regulon structures), without the need to prespecify any of them.

Based on previously-described regulons in *B. subtilis*, we can subsequently determine which regulons naturally emerge in our self-organized expression map. In total, we could identify six clearly-distinct clusters that are associated with known regulons (see S16 and S18 Figure). The first cluster drives the sporulation process and includes the regulons of the four sporulation sigma factors: σ_E, σ_F, σ_K and σ_G[6–8] (cluster 1; S16 and S18 Figure). The second cluster corresponds to the AbrB regulon, involved in the growth transition from exponential to stationary stage[9–11] (cluster 2; S16 and S18 Figure). AbrB also controls the expression of the *eps* operon[12]. The third and fourth clusters belong to the LexA and σ_B regulons, which includes the genes underlying the SOS response and general stress response respectively[13,14] (cluster 3 and 4; S16 and S18 Figure). The fifth cluster includes genes regulated by carbon catabolite repression through CcpA[15] (cluster 5; S16 and S18 Figure). The final cluster contains genes underlying motility, which are part of the σ_D regulon[16,17] (cluster 6; S16 and S18 Figures). The remaining meta-genes did not distinctively fall in any of the previously described regulons (S18 Figure).

In our expression map, we identify fewer regulons than those identified by our regulon-enrichment analysis of the main text. This is partly explained by the fact that some regulons show strongly

overlapping genes, and can therefore not be distinguished in our expression map (SwrA regulon partly overlaps with σ_D; SpoIIID regulon partly overlaps with σ_E; GerE regulon partly overlaps with σ_K; SpoVT regulon partly overlaps with σ_G), but also because genes of some regulons did not cluster on the expression map (e.g. RemA, CodY, DegU, SigI, SinR), either because of their small expression changes or because they showed inconsistent expression changes between lineage 1 and 2. Conversely, we also identified a regulon, the LexA regulon, in our expression map that was not identified in our regulon-enrichment analysis. This is because we performed our regulon-enrichment analysis on gene expression changes in the ancestor only, while our expression map is based on all gene expression samples and therefore accounts for the constitutive expression of the SOS response in lineage 2.

With this expression map at hand, we examined how ancestral and evolved colonies change expression during colony growth. In the ancestor, we see an initial high expression of the sporulation and AbrB regulons. After a few days, these regulons decrease expression, while the regulons of CcpA and σ_D increase expression (see also S3 Figure). The diagonal axis of the expression map, from the upper right to the lower left corner, therefore corresponds to PC1 in the principal component analysis of Figure 3B, as can be visualized by projecting the loading of genes, with regard to PC1, onto the expression map (see S18 Figure, see also S5 Data). The expression changes were consistent with our phenotypic data as well, which showed an initial high fraction of sporulating cells, which declined over time as colony growth progresses (Figure 2).

The expression changes in the evolved colony of lineage 1 largely mimicked those in the ancestor, but started from a lower initial expression of the sporulation and AbrB regulons, and a higher initial expression of the CcpA and σ_D regulons. The expression changes in lineage 2 only partly resembled those in lineage 1. Like lineage 1, lineage 2 initially expressed the sporulation and AbrB regulons at a low level, and it expressed the CcpA and and σ_D regulons at a high level. However, unlike lineage 1, expression of the sporulation regulon first increased, from day 1 to 2, after which it declined (S3 Figure). This explains why the expression trajectory of this colony (week 6) is not fully parallel to PC1 in the principal component analysis of the main text (Figure 3B). In addition, lineage 2 has a constitutive expression of the SOS response (LexA regulon) and a higher expression of the general stress response (σ_B regulon). These expression differences explain the evolutionary divergence between lineage 1 and 2 that is projected along PC2 in the principal component analysis of the main text (Figure 3B and S18).

In summary, the expression maps corroborate our findings in the main text: (1) Expression changes during colony growth in the ancestor involves largely the same genes as those that change expression over evolutionary time in lineage 1 and 2. (2) Expression changes during colony growth in the evolved

colonies are similar to those in the ancestral colony, although having a different starting point (as apparent as well in the PCA in Figure 3B). (3) Evolutionary divergence between lineage 1 and 2 in part results from constitutive expression of the SOS response, which – based on our expression map – also indirectly triggers the general stress response.

References

1. Michna, R. H., Zhu, B., Mäder, U. & Stülke, J. *Subti*Wiki 2.0-an integrated database for the model organism *Bacillus subtilis*. *Nucleic Acids Res* **44**, D654-662 (2016).
2. Mäder, U., Schmeisky, A. G., Flórez, L. A. & Stülke, J. *Subti*Wiki—a comprehensive community resource for the model organism *Bacillus subtilis*. *Nucleic Acids Res* **40**, D1278–D1287 (2012).
3. Zhu, B. & Stülke, J. *Subti*Wiki in 2018: from genes and proteins to functional network annotation of the model organism *Bacillus subtilis*. *Nucleic Acids Res* **46**, D743–D748 (2018).
4. Kohonen, T. Self-organized formation of topologically correct feature maps. *Biol Cybern* **43**, 59–69 (1982).
5. Kohonen, T. *Self-Organizing Maps*. (Springer-Verlag, 2001). doi:10.1007/978-3-642-56927-2.
6. Nicolas, P. *et al.* Condition-dependent transcriptome reveals high-level regulatory architecture in *Bacillus subtilis*. *Science* **335**, 1103–1106 (2012).
7. Eichenberger, P. *et al.* The program of gene transcription for a single differentiating cell type during sporulation in *Bacillus subtilis*. *PLoS Biol* **2**, e328 (2004).
8. Piggot, P. J. & Hilbert, D. W. Sporulation of *Bacillus subtilis*. *Current Opinion in Microbiology* **7**, 579–586 (2004).
9. Zuber, P. & Losick, R. Role of AbrB in Spo0A- and Spo0B-dependent utilization of a sporulation promoter in *Bacillus subtilis*. *J Bacteriol* **169**, 2223–2230 (1987).
10. Hamon, M. A., Stanley, N. R., Britton, R. A., Grossman, A. D. & Lazazzera, B. A. Identification of AbrB-regulated genes involved in biofilm formation by *Bacillus subtilis*. *Mol Microbiol* **52**, 847–860 (2004).
11. Strauch, M. A. *et al.* Abh and AbrB control of *Bacillus subtilis* antimicrobial gene expression. *J Bacteriol* **189**, 7720–7732 (2007).
12. Chumsakul, O. *et al.* Genome-wide binding profiles of the *Bacillus subtilis* transition state regulator AbrB and its homolog Abh reveals their interactive role in transcriptional regulation. *Nucleic Acids Res* **39**, 414–428 (2011).
13. Price, C. W. *et al.* Genome-wide analysis of the general stress response in *Bacillus subtilis*. *Mol Microbiol* **41**, 757–774 (2001).

14. Au, N. *et al.* Genetic composition of the *Bacillus subtilis* SOS system. *J Bacteriol* **187**, 7655–7666 (2005).
15. Singh, K. D., Schmalisch, M. H., Stülke, J. & Görke, B. Carbon catabolite repression in *Bacillus subtilis*: quantitative analysis of repression exerted by different carbon sources. *J Bacteriol* **190**, 7275–7284 (2008).
16. Márquez, L. M. *et al.* Studies of sigma D-dependent functions in *Bacillus subtilis*. *J Bacteriol* **172**, 3435–3443 (1990).
17. Márquez-Magana, L. M. & Chamberlin, M. J. Characterization of the *sigD* transcription unit of *Bacillus subtilis*. *J Bacteriol* **176**, 2427–2434 (1994).
