# Supplementary material for: Pleiotropic hubs drive bacterial surface competition through parallel changes in colony composition and expansion
Source: PLoS Biol. 2023 Oct 16;21(10):e3002338. doi: 10.1371/journal.pbio.3002338 (PMC10578586; doi:10.1371/journal.pbio.3002338)
Supplement: S4 Text — (PDF) [file pbio.3002338.s024.pdf]

S4 Text. Co-activation patterns in *B. cereus*

For comparison to the co-activation patterns of regulators studied in *B. subtilis* (Figure 4), we also examined the co-activation patterns in *B. cereus*. For this, we purposely focus on the replicate population that incurred targeted and independent mutations affecting both sporulation and EPS production (S12 Figure). Like in *B. subtilis*, a large fraction of genes changed expression over the course of our evolution experiment in *B. cereus* (17%, 903/5376; S5 Data). Expression changes during colony growth also resulted from the co-activation of regulators, with two distinct modules corresponding to vegetative growth and dormancy (S19 Figure). Like in *B. subtilis*, this co-activation pattern became partly decoupled in the evolved colonies (S19 Figure), which highlights that mutations affecting global regulators are not required for such decoupling. Instead, expression changes and regulatory decoupling, likely indirectly follow from changes in the colony composition and growth dynamics.
