# Supplementary material for: Pleiotropic hubs drive bacterial surface competition through parallel changes in colony composition and expansion
Source: PLoS Biol. 2023 Oct 16;21(10):e3002338. doi: 10.1371/journal.pbio.3002338 (PMC10578586; doi:10.1371/journal.pbio.3002338)
Supplement: S5 Text — (PDF) [file pbio.3002338.s025.pdf]

S5 Text. Methods

5.1 Evolution experiment, strains and growth conditions

In this study, we build on our previous evolution experiment[1], by performing an in-depth analysis of the populations that evolved the largest increase in colony size. We previously evolved eight *Bacilli* species and strains under a selective regime that favors colony spreading[1]. In brief, for each strain and species, we exposed four replicate populations to the same weekly growth cycle: cells were inoculated in the center of an agar plate (using regular-sized Petri dishes; Ø = 94mm; Ref 633181, Greiner Bio-One) on which they could grow for a week, after which cells from the outermost edge of the colony were transferred to a fresh agar plate. Each agar plate contained 20mL of chemically-defined growth medium, which derived from Fall and colleagues[2], but adjusted to our needs (S1 Table). The medium was solidified using 3% agar (Ref A0949-500, AppliChem) to prevent swimming or swarming motility[3] (for more details on medium preparation see [1]). In total, populations evolved for 11 consecutive growth cycles. To monitor their evolution, we archived each population by making weekly -80°C glycerol stocks. In this study, we use these archived populations to examine the evolution of the two *Bacilli* species, *B. subtilis* and *B. cereus* (S2 Table), which showed the largest increase in colony size over the course of the evolution experiment. We particularly focused on *Bacillus subtilis* subsp. *spizizenii* ATCC 6633, which increased colony size the most[1].

For both *B. subtilis* and *B. cereus*, we isolated two or three clones from each of the weekly-archived populations, which we separately archived and genotyped using whole-genotype sequencing (S3 and S4 Table). We examined the clones by exposing them to the same colony growth cycle as used in the evolution experiment. Specifically, we streaked cells from their glycerol stock on agar plates (as specified above) for overnight growth at 37°C. The next morning cells were scraped from overnight plates with a 10µL inoculation loop (Ref 86.1562.010, Sarstedt) and resuspended in 500µL phosphate-buffered saline (PBS). We diluted the cell suspension to an optical density (λ=600nm) of 0.01 (OD_{600}). From this normalized cell suspension, we pipetted 2µL in the middle of a freshly-made agar plate (for details see van Gestel, Ackermann, and Wagner 2019). The inoculation droplet was subsequently dried, before wrapping the plates in parafilm and incubating them upside down at 37°C for one week. To keep track of colony growth, we imaged colonies each day using an EOS 1200D Canon Camera with EF-S 18-55mm objective for either 6 or 7 days (some clones need more than one day before a clearly opaque colony is visible in the center of the agar plate and can therefore not be imaged on the first day). We also examined the colony composition and gene expression by scraping cells from either the colony center or edge, and either performing flow cytometry analysis or RNA extractions, as further detailed below. Importantly, all phenotyping data (S1, S2, and S4 Data) were newly acquired as part of

this study and are publicly available through either the Supporting Information or online repositories, as specified below.

5.2 Flow cytometry and analysis

Following van Gestel *et al.* 2019[1], we performed flow cytometry by harvesting cells from the colony edge or center using a 10µL inoculation loop (Ref 86.1562.010, Sarstedt), resuspended them in 500µL PBS and normalizing the cells suspension to OD_{600} = 0.001. Cells were stained by adding 5µL of 100x SYBR Green I staining solution, consisting of SYBR Green I stock solution (Ref S7563, Invitrogen, Thermo Fisher Scientific) dissolved in 10mM Tris (pH 8) buffer. Staining was performed at 37°C for 20 minutes in U-bottom 96 deep-well plates (Ref 780261, MASTERBLOCK, Greiner Bio-One). After staining, we analyzed the samples using the BD Accuri C6 flow cytometer (Becton Dickinson, device used from Drinking Water Microbiology group at EAWAG, Dr. Hammes). For each sample, we analyzed 50µL at a maximum flow rate of 66µL/minute. Between all samples we flushed the cytometer with 150µL of filter-sterilized water (0.1µm pore size, Ref SLVV033RS, Millex-VV, Merck Millipore) or 70% ethanol solution to clean the flow cytometer and prevent cross-contamination. All flow cytometry data ($n = 335$, S2 Data) are publicly available in the FlowRepository, accession number FR-FCM-Z6QY.

Following van Gestel *et al.* 2019[1], we analyzed all flow cytometry data in `R` (version 3.4.3), using the `SDMTools`, `alphahull` and `rgl` libraries[4–6]. The gating procedure was separately optimized for both *B. subtilis* and *B. cereus*, using a general gating strategy, as specified in van Gestel *et al.* 2019[1]. In brief, we first discriminated between low and high fluorescent events. Cells, filaments and sporulating cells count as high fluorescent events, because these can be stained by SYBR Green, while (germinating) spores qualify as low fluorescent events, because genomic staining is impossible (SYBR Green cannot penetrate the spore coat). We then distinguish between samples with low and high forward scatter (FSC). Dehydrated spores or forespores (in sporulating cells) are associated with a high refraction index and cause high FSC[7], while germinating spores, cells and filaments are associated with a low FSC. Importantly, because we rely on dehydration of the forespore to detect sporulating cells, genes underlying sporulation can be expressed before we are able to detect sporulating cells using flow cytometry. Finally, we also distinguish between single cells and filaments of different size, by examining the fluorescent width, which correlates with the number of stained genomes and thus the number of cells within a filament. For all flow cytometry experiments, we calibrated the flow cytometer with 3µm fluorescent beads (BD CT&T RUO Beads, Ref 661414, Becton Dickinson). We previously confirmed the accuracy of our gating procedure by using negative control samples (in which cells were filtered out), microscopy, and image flow cytometry[1].

5.3 DNA extractions and sequencing

Following van Gestel *et al.* (2019)[1], we extracted gDNA using the Promega Wizard Genomic DNA Purification Kit (Ref A1120, Promega) using the manufacturer's instructions, with exception for the lysis step, were we resuspended cells in 20mg/mL lysozyme (Ref L6876-1G, Sigma) in 40mM EDTA solution (Ref AM9260G, Ambion) for 60 min at 37°C. We determined the quality of gDNA samples using Qubit dsDNA BR Assay (Ref Q32853, Invitrogen), NanoDrop 1000 UV-VIS Spectrophotometer and a 0.7% agarose (w/v) TBE gel. Library construction and whole-genome sequencing were performed by Oxford Genomics Centre, at the Wellcome Centre for Human Genetics (University of Oxford), using Illumina HiSeq4000 with 150bp paired-end reads. From the 80 gDNA samples, 7 were sequenced as part of our previous study[1], and 73 were newly sequenced (S3 Data). All sequencing data is publicly available on the European Nucleotide Archive (ENA) database, accession number: PRJEB65873.

5.4 RNA extractions and sequencing

For RNA sequencing, we removed cells from the colony (cultured as described above) using a 10µL inoculation loop (Ref 86.1562.010, Sarstedt) and immediately resuspended them in RNAprotect Bacteria Reagent (Ref 76506, QIAGEN). Cells were incubated for 15 min at room temperature (shaking, 700rpm). We removed the supernatant (centrifugation 5000xg, 10min) and resuspended cells in 200µL prepared lysis solution, consisting of 20mg/mL lysozyme (Ref L6876-1G Sigma) and 20µL Protease K solution (Ref 19131, QIAGEN) in TE buffer. Enzymatic lysis was performed for 30 min at room temperature (shaking, 700rpm). We then transferred the cell suspension to a 2mL screw-cap epp tube (Ref 21-403-200, Fisher) with 1.5mL RTL buffer (Ref 79216, QIAGEN), 15uL beta-mercaptoethanol (Z559A, Promega) and 200mg acid-wash beads (Ref G1277-100G, Sigma) for bead beating with the QIAGEN TissueLyzer II (5 min, 30Hz; kindly provided by Gabriella Pessi). We extracted RNA using a standard acid-phenol extraction. Briefly, one volume of phenol/chloroform/isoamyl alcohol (25:24:1, pH4-4.5; Roti-Aqua-P/C/I; Ref X985, Carl Roth, Germany) was added to the cell lysate, mixed and centrifuged. The aqueous phase was carefully transferred to a new epp tube, after which the previous step was repeated. The step was repeated again using one volume of chloroform/isoamyl alcohol (24:1), after which the aqueous phase was transferred to a new epp tube, 1/10 volume 3M Na-Acetate (pH 5.2) and 1.5 volumes of isopropanol were added, and RNA was allowed to precipitate overnight at -20°C. The next day, we washed the RNA pellet twice using 70% EtOH and removed genomic DNA, using Ambion Turbo DNA-free DNase (following manufacturer's instructions; ref AM1907, Life Technologies). We then precipitated the RNA again and eluted this in nuclease-free water (Ref P1193, Promega). We determined the RNA yield and quality using Qubit RNA broad package (Ref Q10210, Invitrogen), NanoDrop 1000 UV-VIS Spectrophotometer and an agarose

gel (2% agarose (w/v) with 6% formaldehyde (v/v) in 20mM MOPS, 5mM Na-Acetate, 1mM EDTA buffer). An additional quality check (Agilent TapeStation), ribosomal depletion (using NEB bacterial rRNA probes), library construction and sequencing were performed by Oxford Genomics Centre, at the Wellcome Centre for Human Genetics (University of Oxford), using Illumina RNA seq NovaSeq6000 with 150bp paired-end reads. Since we had a total of 84 RNA samples, sequencing was done using two lanes, resulting in 168 paired-end read files (S4 Data), which are publicly available on the European Nucleotide Archive (ENA) database, accession number: PRJEB65873.

5.5 Data analyses

5.5.1 Colony data

We processed all colony images ($n = 1389$ images, see S1 Data) using custom-made image analysis software (Github Repository https://github.com/jordivangestel/PLoS-Biology-2023 and Zenodo http://doi.org/10.5281/zenodo.8360460), with easy-to-use general user interface (S20 Figure), which we programmed in Matlab 2016 (MathWorks). In brief, our software first detects the Petri-dish based on a simple segmentation procedure. In this first step, the software scales the image such that it only includes the region inside the Petri-dish, thereby ignoring everything that falls outside of this region (S20B Figure). Petri-dish should be imaged on a dark background, preferably non-reflective black paper, for automatic segmentation to work effectively. In the rare case that segmentation fails, parameters can be manually adjusted, and the segmentation procedure can be visualized to optimize parameter settings (S20A Figure). After segmenting the Petri-dish, the region inside the Petri-dish is analyzed to detect the colony outline. This is starts with a simple segmentation step where the colony is distinguished from the agar plate. This segmentation step also relies on a few parameter settings, which – if needed – can be manually adjusted. The binary image does not by itself provide an optimized outline. Therefore, in the final step, we use the binary image to draw an outline around the colony, which is defined by coordinates of regularly-spaced points (S20C Figure). The coordinates of these points are further optimized in a refinement step that makes use of local pixel intensities. That is, for each point on the outline, we extract the pixel values that lie perpendicular to the outline (S20D Figure). These pixel values are expected to follow a sigmoidal curve, with higher pixel values towards the colony center and lower values away from the colony (S20E Figure). In the ideal case, the outline matches the inflection point of the sigmoidal curve. If this is not the case, the outline will be refined (S20F Figure). The parameter conditions that are used to refine the outline can be adjusted manually. Also, in the rare case that automatic detection of the outline fails, one can also draw the outline manually. The image analysis software is provided on our Github Repository

(https://github.com/jordivangestel/PLoS-Biology-2023) and Zenodo (http://doi.org/10.5281/zenodo.8360460).

5.5.2 Genotypic data

In total, we genotyped 80 clones with whole-genome sequences (S3 Data): for *B. subtilis* lineage 1 and 2 we sequenced two or three clones for each of the weekly-archived cell populations in the evolution experiment. For the other *B. subtilis* lineages as well as the *B. cereus* lineage, we sequenced clones that were associated with changes in the colony size during the evolution experiment as well as three clones from the end of the evolution experiment. We used `Breseq v0.32.0`[8,9] to map the sequence reads against the associated reference genomes and detect genetic changes between the ancestral and evolved clones. All mutations detected by `Breseq` were confirmed through visual inspection using Integrated Genome Browser, `IGVTools 2.4.19`[10].

5.5.3 RNA data

We extracted RNA for 9 genetically distinct clones (from both *B. subtilis* and *B. cereus*), from either the colony center or edge at day 1, 2, 4 or 7 in the weekly growth cycle. Per condition, we included two to three biological replicates, which – together with the technical replicates – resulted in 84 RNA extractions. To assure sufficient read depth, two Illumina NovaSeq6000 sequence lanes were used for sequencing, resulting in 168 expression profiles (paired-end reads), two for each RNA extraction (see S4 Data). These expression profiles were processed using the following steps. We first trimmed reads using `Trimmomatic-0.39`[11], to remove adapters, using the following settings: `ILLUMINACLIP:TruSeq3-PE.fa:2:30:10 LEADING:10 TRAILING:10 SLIDINGWINDOW:5:15 MINLEN:35`. Then, we confirmed read quality using both `FastQC`[12] and `MultiQC`[13]. Next, we aligned the reads using `Bowtie2`[14]. The resulting BAM files were processed using `SAMtools-1.7`[15]. Finally, we determined read counts for each gene using `HTSeq`[16] (`-m intersection-nonempty`) in `Python v3.6`. We also used the aligned reads (i.e. SAM files) to generate wiggle files (`IGVTools 2.4.19`[10], which were subsequently used to predict transcription start sites and endings, using `Parseq`[17].

For further downstream processing, we used `R v3.6.0`. We first combined the count data of the expression profiles that were associated with the same RNA extraction (given that sequencing was done on two separate Illumina lanes). We removed genes with a low read coverage, with an average of less than 10 read counts. 94.4% (3814/4039) of the *Bacillus subtilis* genes and 90.2% (4850/5376) of the *Bacillus cereus* genes passed our filter criteria (more lenient filter criteria give nearly identical results). Counts were normalized using `DESeq2`[18] and log transformed, with a pseudo-count of 1, for the principal component analysis in Figure 3B (`normTransform` function in `DESeq2`). Differentially

expressed genes were determined using a Wald test for the GLM coefficients (DESeq function in DESeq2) using a full model design (~time+genotype+time:genotype) in both lineage 1 or 2 in *B. subtilis* and lineage 1 *in B. cereus*. To rank the differentially expressed genes (adjusted $p < 0.01$) according to their log-fold changes, we applied log-fold change shrinkage based on apeglm[19], using the lfcShrink function in DESeq2. For our purpose, we considered genes with a significant (adjusted $p < 0.01$) two-fold change in expression between the ancestral and evolved colonies for downstream analysis, as well as genes that significantly changed expression during a colony growth cycle in the ancestor (with a $> 10\%$ change in expression a day). The normalized count data of all samples as well as the full lists of differentially expressed genes are provided in Data S5.

For the Venn diagram in Figure 3A, we compare the overlap between three sets of differentially expressed genes: (1) genes that significantly change expression during colony growth in the ancestor, using the above criteria ($> 10\%$ expression change a day with an adjusted $p < 0.01$), (2) genes that significantly change expression between the ancestral and evolved genotype in lineage 1 (i.e., isolate from week 5), using a full model design (two-fold change in gene expression between genotypes with an adjusted $p < 0.01$), and (3) genes that significantly change expression between the ancestral and evolved genotype in lineage 2 (i.e., isolated from week 6), using a full model design (two-fold change in gene expression between genotypes with an adjusted $p < 0.01$). There is a significant overlap between these different gene sets, based on the Fisher's exact test (fisher.test in R). The gene sets are provided in Data S5. Although our selection criteria for assigning differentially expressed genes will affect the Venn diagram shown Figure 3A, they do not impact our main conclusions. That is, also when considering all expression changes, and not only those deemed significant, we find that genes changing expression during colony development in the ancestor likewise change expression during our evolution experiment (Figure 3B).

To examine co-expression patterns of genes in Text S3, we made use of Kohonen's self-organizing map[20], which is an unsupervised machine learning approach that allows us to map genes on a two dimensional hexagonal plane according to the similarity in expression across all RNA-seq samples (S16 Figure). This machine learning approach results in both dimensionality reduction as well as gene clustering[21]. We trained the self-organizing map based on the log-ratios of normalized gene expression counts over their geometric mean count across all samples. For generating the self-organizing map, we made use of the kohonen package[22,23] in R with the following parameter settings (in the som function): 20x20 hexagonal grid, rlen=1000, alpha=[0.1,0.01].

For the regulon enrichment analysis, we examined the 40 largest regulons as described by the *Subti*wiki database on May 4th 2020[24–26]. We determined which of these regulons are enriched among

the differentially expressed genes in the ancestor, using a two-sided Fisher's exact test (`fisher.test` in `R`). p values were adjusted for multiple testing using the Benjamini-Yekutieli procedure[27] (`method="BY"` for `p.adjust` in `R`). In total, we identified 18 enriched regulons: one phage regulon (associated with the phage sigma factor Xpf) and 17 innate regulons. For our downstream analysis we ignored the phage regulon. For the 17 innate regulons, we determined the activity of the corresponding regulators, following the methods described by Arrieta-Ortiz and colleagues[28]. That is, we inferred the regulatory activity based on the type of regulation and the expression of the downstream genes: a repressor is assumed to be active when genes in its regulon have low expression and an activator is assumed to be active when genes in its regulon have high expression. We subsequently examined co-activity patterns by generating a correlogram (`corrplot` function in `R`) based on the pairwise Pearson correlations between the activities of regulators during the colony growth cycle in either the ancestor (Figure 4A) or evolved colonies in lineage 1 (Figure 4B) or 2 (Figure 4C). For the ancestor, we show the Pearson's correlation coefficient and R square in Data S5. We used hierarchical clustering (`hclust` function in `R`) to identify the modular organization of the correlogram, which separates the regulators active during vegetative growth from those active towards dormancy.

An R script for analyzing any of the gene expression changes is available in our Github repository (https://github.com/jordivangestel/PLoS-Biology-2023) and Zenodo (http://doi.org/10.5281/zenodo.8360460).

References

1. van Gestel, J., Ackermann, M. & Wagner, A. Microbial life cycles link global modularity in regulation to mosaic evolution. *Nat Ecol Evol* **3**, 1184–1196 (2019).
2. Fall, R., Kearns, D. B. & Nguyen, T. A defined medium to investigate sliding motility in a *Bacillus subtilis* flagella-less mutant. *BMC Microbiol* **6**, 31 (2006).
3. Kearns, D. B. A field guide to bacterial swarming motility. *Nat Rev Micro* **8**, 634–644 (2010).
4. Pateiro-López, B. & Rodrıguez-Casal, A. Generalizing the convex hull of a sample: the R package alphahull. *Journal of Statistical software* **34**, 1–28 (2010).
5. VanDerWal, J., Falconi, L., Januchowski, S., Shoo, L. & Storlie, C. SDMTools: tools for processing data associated with species distribution modelling exercises. *R package version 1* (2014).
6. Adler, D. *et al.* Rgl: 3D visualization using OpenGL. *R package version 0.95* (2016).
7. Ross, K. F. A. & Billing, E. The water and solid content of living bacterial spores and vegetative cells as indicated by refractive index measurements. *Microbiology* **16**, 418–425 (1957).

8. Barrick, J. E. *et al.* Identifying structural variation in haploid microbial genomes from short-read resequencing data using *breseq*. *BMC Genomics* **15**, 1039 (2014).
9. Deatherage, D. E. & Barrick, J. E. Identification of mutations in laboratory-evolved microbes from next-generation sequencing data using *breseq*. in *Engineering and Analyzing Multicellular Systems: Methods and Protocols* (eds. Sun, L. & Shou, W.) 165–188 (Springer New York, 2014).
10. Thorvaldsdóttir, H., Robinson, J. T. & Mesirov, J. P. Integrative Genomics Viewer (IGV): high-performance genomics data visualization and exploration. *Briefings in Bioinformatics* **14**, 178–192 (2013).
11. Bolger, A. M., Lohse, M. & Usadel, B. Trimmomatic: a flexible trimmer for Illumina sequence data. *Bioinformatics* **30**, 2114–2120 (2014).
12. Andrews, S. FastQC. https://github.com/s-andrews/FastQC. (2021).
13. Ewels, P., Magnusson, M., Lundin, S. & Käller, M. MultiQC: summarize analysis results for multiple tools and samples in a single report. *Bioinformatics* **32**, 3047–3048 (2016).
14. Langmead, B. & Salzberg, S. L. Fast gapped-read alignment with Bowtie 2. *Nat Methods* **9**, 357–359 (2012).
15. Li, H. *et al.* The sequence alignment/nap format and SAMtools. *Bioinformatics* **25**, 2078–2079 (2009).
16. Anders, S., Pyl, P. T. & Huber, W. HTSeq—a Python framework to work with high-throughput sequencing data. *Bioinformatics* **31**, 166–169 (2015).
17. Mirauta, B., Nicolas, P. & Richard, H. Parseq: reconstruction of microbial transcription landscape from RNA-Seq read counts using state-space models. *Bioinformatics* **30**, 1409–1416 (2014).
18. Love, M. I., Huber, W. & Anders, S. Moderated estimation of fold change and dispersion for RNA-seq data with DESeq2. *Genome Biology* **15**, 550 (2014).
19. Zhu, A., Ibrahim, J. G. & Love, M. I. Heavy-tailed prior distributions for sequence count data: removing the noise and preserving large differences. *Bioinformatics* **35**, 2084–2092 (2019).
20. Kohonen, T. *Self-Organizing Maps*. (Springer-Verlag, 2001). doi:10.1007/978-3-642-56927-2.
21. Asan, U. & Ercan, S. An Introduction to Self-Organizing Maps. in *Computational Intelligence Systems in Industrial Engineering: With Recent Theory and Applications* (ed. Kahraman, C.) 295–315 (Atlantis Press, 2012). doi:10.2991/978-94-91216-77-0_14.
22. Wehrens, R. & Buydens, L. M. C. Self- and super-organizing maps in R: the Kohonen package. *Journal of Statistical Software* **21**, 1–19 (2007).
23. Wehrens, R. & Kruisselbrink, J. Flexible self-organizing maps in Kohonen 3.0. *Journal of Statistical Software* **87**, 1–18 (2018).

24. Michna, R. H., Zhu, B., Mäder, U. & Stülke, J. *Subti*Wiki 2.0-an integrated database for the model organism *Bacillus subtilis*. *Nucleic Acids Res* **44**, D654-662 (2016).
25. Mäder, U., Schmeisky, A. G., Flórez, L. A. & Stülke, J. *Subti*Wiki—a comprehensive community resource for the model organism *Bacillus subtilis*. *Nucleic Acids Res* **40**, D1278–D1287 (2012).
26. Zhu, B. & Stülke, J. *Subti*Wiki in 2018: from genes and proteins to functional network annotation of the model organism *Bacillus subtilis*. *Nucleic Acids Res* **46**, D743–D748 (2018).
27. Benjamini, Y. & Yekutieli, D. The control of the false discovery rate in multiple testing under dependency. *Annals of Statistics* **29**, 1165–1188 (2001).
28. Arrieta-Ortiz, M. L. *et al.* An experimentally supported model of the *Bacillus subtilis* global transcriptional regulatory network. *Mol Syst Biol* **11**, 839 (2015).
