# Supplementary material for: Pleiotropic hubs drive bacterial surface competition through parallel changes in colony composition and expansion
Source: PLoS Biol. 2023 Oct 16;21(10):e3002338. doi: 10.1371/journal.pbio.3002338 (PMC10578586; doi:10.1371/journal.pbio.3002338)
Supplement: S1 Table — (PDF) [file pbio.3002338.s026.pdf]

S1 Table. Chemically-defined growth medium

#	Chemical compound	Chemical Formula	Concentration	Company
1	Sodium Phosphate Buffer (pH 7.3)	$Na_2HPO_4 + NaH_2PO_4$	2.5mM	Fluka #71645 + Fluka #71500
2	Glycerol	$C_3H_8O_3$	5.4mM (=0.05%)	AppliChem #A3739
3	Glutamate	$C_5H_8NNaO_4$	30mM (=0.5%)	Sigma-Aldrich #G5889
4	MOPS (pH 7)	$C_7H_{15}NO_4S$	0.01M	AppliChem #A2947
5	Manganese(II) chloride	$MnCl_2$	0.05mM	Sigma-Aldrich #31422
6	Magnesiumchloride	$MgCl_2$	2mM	Fluka #63065
7	Ferric chloride	$FeCl_3$	0.05mM	Sigma-Aldrich #236489
8	Calciumchloride	$CaCl_2$	0.7mM	Sigma-Aldrich #C3881
9	Potassium chloride	KCl	0.1mM	Sigma-Aldrich #60130
10	Zinc chloride	$ZnCl_2$	1uM	Merck #7815
