# Supplementary material for: Pleiotropic hubs drive bacterial surface competition through parallel changes in colony composition and expansion
Source: PLoS Biol. 2023 Oct 16;21(10):e3002338. doi: 10.1371/journal.pbio.3002338 (PMC10578586; doi:10.1371/journal.pbio.3002338)
Supplement: S2 Table — (PDF) [file pbio.3002338.s027.pdf]

S2 Table. Strains

strain	Source	RefSeq accession
Bacillus subtilis subsp. *spizizenii* ATCC 6633	Kolter lab	GCF_000177595.1
Bacillus cereus ATCC 10987	BGSC	GCF_000008005.1
