# Supplementary material for: Pleiotropic hubs drive bacterial surface competition through parallel changes in colony composition and expansion
Source: PLoS Biol. 2023 Oct 16;21(10):e3002338. doi: 10.1371/journal.pbio.3002338 (PMC10578586; doi:10.1371/journal.pbio.3002338)
Supplement: S3 Table — (PDF) [file pbio.3002338.s028.pdf]

S3 Table. *Bacillus subtilis* subsp. *spizizenii* ATCC 6633

Locus tag / gene name	Ortholog*	location	mutation	Week 1.1	Week 1.2	Week 2.1	Week 2.2	Week 3.1	Week 3.2	Week 4.1	Week 4.2	Week 5.1	Week 5.2	Week 6.1	Week 6.2	Week 7.1	Week 7.2	Week 8.1	Week 8.2	Week 9.1	Week 9.2	Week 10.1	Week 10.2	Week 11.1	Week 11.2	Week 11.3
**Lineage 1**																										
EO946_RS00220	*ricT*	41,470	Q43* (CAG→TAG)				•	•	•	•	•	•	•	•	•	•	•	•	•	•	•	•	•	•	•	•
EO946_RS17240	*epsK*	3,335,653	(A)7→8									•		•	•	•	•	•	•	•	•	•	•	•	•	•
EO946_RS15020	NA	2,917,728	V208V (GTG→GTA)																			•	•	•	•	•
bshC / EO946_RS08090	*bshC* / *ytrE*	1,546,565	(T)9→8																					•	•	•
spo0A	*spo0A*	2,392,265	(GTTGGTAGGTTTGGCT)1→2		•																					
EO946_RS07485	*mcpC*	1,431,292	G176E (GGG→GAG)		•																					
pnp	*pnpA*	1,708,765	R531R (AGA→AGG)			•																				
fba	*iolJ*	3,914,609	R15W (CGG→TGG)						•																	
EO946_RS10365	NA	2,063,307	L103L (CTG→CTA)							•	•															
**Lineage 2**																										
EO946_RS09000 / *rny*	*rny*	1,735,013	(A→T)			•	•	•	•	•	•	•	•	•	•	•	•	•	•	•	•	•	•	•	•	•
EO946_RS20275 / EO946_RS20280	*qdol*	3,940,915	(C→T)			•	•	•	•	•	•	•	•	•	•	•	•	•	•	•	•	•	•	•	•	•
EO946_RS16590	*liaF*	3,204,792	Δ63 bp					•			•	•	•	•	•	•	•	•	•	•	•	•	•	•	•	•
lexA	*lexA*	1,882,071	P25L (CCG→CTG)											•	•	•	•	•	•	•	•	•	•	•	•	•
EO946_RS05260	*bmrD*	1,020,731	L396L (TTG→CTG)																		•	•	•	•	•	•
EO946_RS09125	*pksJ*	1,767,043	R1851Q (CGG→CAG)														•				•	•	•	•	•	•
yugI / EO946_RS15675	*yugI*	3,031,616	(A→G)																					•	•	•
polX	*polX*	2,734,856	P203S (CCG→TCG)	•	•																					
EO946_RS17250	*epsI*	3,338,118	R119C (CGC→TGC)	•	•																					
EO946_RS19755	NA	3,838,423	G180G (GGA→GGG)	•	•																					
EO946_RS20565	*rplI*	4,003,373	(CTTG)2→1							•																
EO946_RS05245	*nhaX*	1,017,182	R54W (AGG→TGG)							•																
yneA	*yneA*	1,882,448	A52V (GCT→GTT)													•										
EO946_RS07705	*fruA*	1,474,849	H595L (CAC→CTC)															•								
argJ	*argJ*	1,166,051	A275V (GCC→GTC)																			•				
**Lineage 3**																										
EO946_RS17265	*epsF*	3,341,441	C117* (TGC→TGA)					•																•	•	•
**Lineage 4**																										
EO946_RS16965	*spaB*	3,280,509	(T)8→9			•												•						•	•	•
EO946_RS17275	*epsD*	3,343,428	H112R (CAC→CGC)															•						•	•	•

*Orthologous genes (based on bidirectional-best BLAST hits with *B. subtilis 168*) and functions (based on SubtiWiki database): *argJ* = biosynthesis of arginine; *bmrD* = multidrug ABC transporter (ATP-binding protein), also involved in the signaling pathway to activate KinA at the onset of sporulation; *bshC* = Bacillithiol biosynthesis cysteine-adding enzyme; *epsK, epsI, epsF, epsD* = extracellular polysaccharide production; *fba* = class II fructose-1,6-bisphosphate aldolase; *fruA* = fructose uptake and phosphorylation; *lexA* = transcriptional repressor of the SOS regulon; *liaF* = control of LiaR involved in membrane stress and antibiotic resistance; *mcpC* = membrane-bound chemotaxis receptor for proline; *nhaX* = general stress protein; *pksJ* = Bacillaene synthesis; *pnpA* = polynucleotide phosphorylase, RNase, involved in double-strand break repair; *polX* = DNA polymerase X, involved in DNA repair; *qdol* = Fe-containing quercetin 2,3-dioxygenase; *ricT* = subunit of the regulatory iron-sulfur containing RicA-RicF-RicT complex, required for RNase Y dependent maturation of polycistronic mRNAs, control of the phosphorelay, required for the achieving a sufficient level of Spo0A-P for sporulation initiation; *rny* = RNase Y, 5' end sensitive endoribonuclease, involved in the degradation/processing of mRNA, part of the putative RNA degradosome; *rplI* = ribosomal protein L9; *spaB* = Subtilin biosynthesis protein; *spo0A* = phosphorelay regulator, initiation of sporulation; *yneA* = inhibitor of cell division during SOS response (mutation in LysM peptidoglycan binding domain involved in inhibiting cell division, resulting in filamentous growth; shares promoter directly with *lexA*); *ytrE* = ABC transporter (ATP-binding protein) involved in resistance to cell wall inhibitors; *yugI* = similar to domains in *E. coli* ribosomal protein S1, subject to stringent response.
