# Supplementary material for: Pleiotropic hubs drive bacterial surface competition through parallel changes in colony composition and expansion
Source: PLoS Biol. 2023 Oct 16;21(10):e3002338. doi: 10.1371/journal.pbio.3002338 (PMC10578586; doi:10.1371/journal.pbio.3002338)
Supplement: S4 Table — (PDF) [file pbio.3002338.s029.pdf]

S4 Table. *Bacillus cereus* ATCC 10987

Locus tag / gene name	Ortholog*	location	mutation	Week 1.1	Week 1.2	Week 2.1	Week 2.2	Week 3.1	Week 3.2	Week 4.1	Week 4.2	Week 5.1	Week 5.2	Week 6.1	Week 6.2	Week 7.1	Week 7.2	Week 8.1	Week 8.2	Week 9.1	Week 9.2		Week 10.1	Week 10.2	Week 11.1	Week 11.2	Week 11.3
Lineage 1																											
BCE_RS00270/BCE_RS00275	*ridA/spoVG*	50,519	Insertion of IS4-like element					•	•			•													•	•	•
BCE_RS10175	NA	1,992,511	F407V (TTT→GTT)									•													•	•	•
BCE_RS25840	*epsF*	4,978,673	(T)7→6									•													•	•	•
BCE_RS15440	*ftsX*-like	3,006,018	(CTTT)3→4																						•	•	•
BCE_RS04685	NA	914,427	Δ30 bp																								•
BCE_RS17670	NA	3,435,949	F139V (TTT→GTT)									•															
Lineage 2																											
spo0A	*spo0A*	3,956,556	T86I (ACA→ATA)											•	•										•	•	•
BCE_RS17475	NA	3,401,114	H315Y (CAT→TAT)																							•	•
BCE_RS01280	NA	236,214	Q96* (CAA→TAA)												•												
BCE_RS14970 / BCE_RS28725	NA	2,911,893	(T→C)																						•		
Lineage 3																											
BCE_RS05175 / BCE_RS29525	*yjdF* / NA	1,017,579	(A)8→7																						•	•	•
BCE_RS10180	*yheN*	1,993,475	V240V (GTG→GTA)																						•	•	•
BCE_RS25880	*ptkA*	4,987,198	Δ21 bp																						•	•	•
Lineage 4																											
BCE_RS25810	*ytcB*	4,971,829	E56* (GAA→TAA)					•																	•	•	•
BCE_RS19135	*ylaK*	3,748,046	+C																						•	•	•

*Orthologous genes (based on bidirectional-best BLAST hits with *B. subtilis 168*) and functions (based on SubtiWiki database): *epsF* = extracellular polysaccharide production; *ftsX* = permeases required for activation of *spo0A* and thus sporulation; *ptkA* = protein tyrosine kinase, paralog to *epsB* involved in regulating extracellular polysaccharide production in biofilm formation; *spoVG* = involved in asymmetric septation in sporulation; *spo0A* = phosphorelay regulator, initiation of sporulation; *ylaK* = similar to phosphate starvation inducible protein *PhoH*, expressed during sporulation; *yheN* = putative polysaccharide deacetylase; *ytcB* = putative UDP-glucose epimerase, expressed during sporulation (regulated by SigK and GerE).
